# Sensitization to molecular dog allergens in an adult population: Results from the West Sweden Asthma Study

**DOI:** 10.1111/cea.14216

**Published:** 2022-09-01

**Authors:** Saliha Selin Özuygur Ermis, Magnus P. Borres, Rani Basna, Linda Ekerljung, Carina Malmhäll, Emma Goksör, Göran Wennergren, Madeleine Rådinger, Jan Lötvall, Bo Lundbäck, Hannu Kankaanranta, Bright I. Nwaru

**Affiliations:** ^1^ Krefting Research Centre Institute of Medicine, Sahlgrenska Academy, University of Gothenburg Gothenburg Sweden; ^2^ Department of Respiratory Medicine Dokuz Eylul University Izmir Turkey; ^3^ ImmunoDiagnostics Thermo Fisher Scientific Uppsala Sweden; ^4^ Department of Maternal and Child Health Uppsala University Uppsala Sweden; ^5^ Department of Pediatrics University of Gothenburg, Queen Silvia Children's Hospital Gothenburg Sweden; ^6^ Faculty of Medicine and Life Sciences University of Tampere Tampere Finland; ^7^ Department of Respiratory Medicine Seinäjoki Central Hospital Seinäjoki Finland; ^8^ Wallenberg Centre for Molecular and Translational Medicine Institute of Medicine, University of Gothenburg Gothenburg Sweden

**Keywords:** component resolved diagnostics, dog allergy, dog dander sensitization, dog molecular allergen components, furry animal allergy, Can f 1, Can f 5

## Abstract

**Background:**

As the prevalence of dog allergy rises, component resolved diagnosis might improve the diagnosis, understanding of the clinical outcomes and the effectiveness of immunotherapy. Considering the paucity of data in adults, the current study characterized the patterns of sensitization to dog molecular allergens in an adult population.

**Methods:**

Data were derived from the West Sweden Asthma Study, a population‐based and representative sample of adults from western Sweden. Of the 2006 subjects clinically examined, 313 participants sensitized to whole dog allergen extract were measured for specific immunoglobulin E (sIgE) levels to Can f 1, Can f 2, Can f 3, Can f 4, Can f 5 and Can f 6 using ImmunoCAP™. Polysensitization was defined as sensitization to **≥**3 components. Overlapping sensitization was defined as having concomitant sensitization to at least two dog molecular allergen families (lipocalin, albumin or prostatic kallikrein).

**Results:**

Of 313, 218 (70%) subjects tested positive to at least one dog allergen component. Sensitization to Can f 1 (43%) was the most common, followed by Can f 5 (33%) among molecular allergens, while sensitization to lipocalins (56%) was the most common among component families. Polysensitization was found in 22% of all participants and was more common in participants with than in those without asthma. Subjects with asthma were less likely to be monosensitized to Can f 5 than those without asthma. Subjects with asthma had higher IgE levels of Can f 3, Can f 4 and Can f 6 than those without asthma. Overlapping sensitizations also differed between those with asthma and allergic rhinitis and those without.

**Conclusion:**

Increased knowledge about the sensitization patterns of dog allergen components can aid in defining their role in asthma and rhinitis. In complex clinical cases of dog allergy, a detailed analysis of dog allergen components can provide additional information on the nature of sensitization.

**Key Message**

- Can f 1 (43%) and Can f 5 (33%) were the most commonly sensitized single components.
- Subjects with asthma had higher IgE levels of Can f 3, Can f 4 and Can f 6.
- Polysensitization rate was 22% and more prevalent in subjects with asthma.

## 1 INTRODUCTION

The prevalence of sensitization to aeroallergen and pet allergy continues to increase globally in line with increasing numbers of pet ownership.^1^ Domestic dogs (*Canis familiaris*) constitute one of the commonest sources of indoor allergens.^2^, ^3^ Dog dander, hair, urine and saliva are the sources of various allergenic molecules of dogs, which easily become airborne.^4^ While dog allergy is relatively common among subjects with airborne allergies, the prevalence of allergic sensitization to dogs has also significantly increased from 13% to 25% between 1994 and 2009.^3^, ^5^, ^6^, ^7^, ^8^ On the other hand, the prevalence of dog sensitization varies across countries, regions and methods of detection. A Swiss study revealed that the prevalence of dog sensitization, defined by the skin prick test (SPT), was 3%, whereas it was 13% in Finland also defined by SPT positivity.^9^, ^10^ Simpson et al^11^ found that 10% of adults in Manchester, UK, were sensitized to dog dander according to SPT. As current commercial dog extracts have not been standardized and show great variance in SPT solutions, further diagnostic tools are needed to define allergic sensitization.^5^, ^12^

During the last decade, developments in molecular‐based diagnostic techniques, component resolved diagnosis (CRD), are improving the diagnosis of pet allergy.^4^, ^13^, ^14^ The CRD approaches are hoped to overcome the limitations of conventional diagnostic techniques, such as SPT and immunoassays of serum specific immunoglobulin E (sIgE), through improved specificity and sensitivity of the test, better prediction of the severity of the allergic reaction, and detection of cross‐reaction of allergens.^4^, ^13^, ^14^, ^15^ As IgE antibodies to allergen molecules may vary from patient to patient, CRD approaches may help to compile patient‐tailored risk profiles to specific allergens.^4^, ^13^, ^14^ They can also help to distinguish a primary sensitization from a cross‐sensitization to a higher extent than whole allergen extracts will do because they quantify sensitization to the individual allergen proteins that may be responsible for allergic reactions.^4^, ^13^, ^14^

Out of eight dog molecular allergens listed in the WHO/IUIS Allergen Nomenclature database, six are currently available for clinical use, subdivided into three major families: the lipocalin protein family, albumin and prostatic kallikrein.^15^, ^16^, ^17^, ^18^ Lipocalins constitute four allergen components: Can f 1, Can f 2, Can f 4 and Can f 6; while albumins consist of Can f 3.^19^, ^20^, ^21^, ^22^, ^23^, ^24^ The third major family, prostatic kallikrein, includes only Can f 5, a protein that is found only in male dogs.^13^ The current knowledge about the clinical implications of dog molecular allergen components primarily comes from the paediatric population. Children sensitized to Can f 1 and Can f 2 have a greater risk of severe asthma than those not sensitized to these components.^2^, ^6^ Moreover, sensitization to lipocalins is related to dog allergy, while Can f 5 monosensitization was not detected in children.^16^ On the other hand, there is a paucity of data among adults about dog molecular components. The knowledge of dog molecular allergen components and their impact on asthma and rhinitis in adults warrants increased investigations. To contribute to knowledge on molecular dog allergens in adults, this study provides a detailed characterization of sensitization to dog allergen components in a Swedish adult population‐representative sample.

## 2 METHODS

### 2.1 Study participants

The study data were derived from an ongoing population‐based longitudinal study, the West Sweden Asthma Study (WSAS). Details of the study procedures have been described elsewhere.^25^, ^26^ We sent out 30,000 postal questionnaires to randomly selected 16–75‐year‐old adults living in the Västra Götaland region of western Sweden in 2008. We received back 18,087 completed questionnaires (response rate of 62%) after correction for untraced and subjects who had died. Of the respondents, 2000 were randomly selected and invited to clinical investigations, of which 1172 participated. Additionally, those who reported having asthma based on the initial questionnaire (*n* = 1524) were also invited to the clinical investigations; of which 834 participated. In total, 2006 subjects took part in the clinical examinations, and 1872 of these gave blood sample for the measurement of IgE to a mix of aeroallergens (Phadiatop™). Written informed consent was obtained from all study participants. Of these subjects, 313 (17%) were sensitized to dog dander based on IgE positivity (≥0.35 kU_A_/L) and thus formed the sample included in the current study (Figure S1).

### 2.2 Molecular allergen components to dog and sensitization patterns

Following the analysis of IgE levels against a mix of aeroallergens (Phadiatop™) from serum, subjects with titers of ≥0.35 kU_A_/L were then measured for IgE antibody levels against specific dog allergen components (Can f 1, Can f 2, Can f 3, Can f 4, Can f 5 and Can f 6) using ImmunoCAP™ (Phadia AB). Values of IgE ≥ 0.35 kU_A_/L for an individual component were considered positive. In addition to each specific allergen component, we also defined sensitization to the major families of dog allergen components: lipocalin (sensitization to Can f 1, Can f 2, Can f 4 or Can f 6); prostatic kallikrein (sensitization to Can f 5) and serum albumin (sensitization to Can f 3). We also defined concomitant sensitization among the three allergen families; monosensitization to lipocalin; monosensitization to prostatic kallikrein and polysensitization (sensitization to 3 or more individual components).

### 2.3 Participants' background characteristics

The following demographic characteristics were collected: age, smoking, body mass index (BMI), degree of urbanization, exposure to dust/fumes at the place of work, raised on a farm, highest education attained, family history of allergy or asthma and current dog ownership.^25^, ^26^ We also collected information on the current asthma and allergic rhinitis status of the participants. *Current asthma* was defined as affirmative answers to either of the two following questions: “Have you ever had asthma?” or “Have you ever been diagnosed as having asthma by a physician?” in combination with any of the following: use of asthma medication, recurrent wheeze or attacks of shortness of breath during the last 12 months. *Current rhinitis* was defined based on affirmative answers to either of the following questions: “Do you have sneezing, runny nose, or nasal block without having a cold?” or “During the last 12 months, have you used medicines for hay fever or other problems of rhinitis, such as the runny nose or nasal blocking without having a cold?”.

### 2.4 Statistical analyses

The Pearson chi‐square test was used to examine differences between categorical variables. Mann–Whitney *U* test was used to examine differences in median IgE levels of each dog allergen component and distributed by the sensitization patterns, allergen component families and asthma and allergic rhinitis status. We used Venn diagrams to describe the overlap in sensitization among the dog allergen components. Sensitization overlaps were stratified by current dog ownership, presence of asthma and/or allergic rhinitis, obesity (BMI ≥ 30) and smoking status. In all statistical tests, statistical significance was taken as *p* < .05. Analyses were carried out using GraphPad Prism Version 9.0.0, GraphPad Software, and IBM SPSS Statistics for Windows, Version 22.0. IBM Corp.

## 3 RESULTS

### 3.1 Characteristics of study participants

Three hundred and thirteen subjects who were sensitized to dog dander (IgE ≥ 0.35kU/L) were included in the study (Figure S1). The mean age was 43.0 years and 52.7% of participants were females. Of the participants, 20.1% were obese; 14.7% currently owned a dog; 70.6% had current asthma while 78.3 % had current allergic rhinitis (Table S1). Of 313, 69.6% (*n* = 218) were sensitized to at least one molecular allergen component (Table 1). There were no differences between individuals sensitized to at least one molecular component and those not sensitized regarding the participants' characteristics except subjects sensitized to at least one component had higher concomitance of asthma and allergic rhinitis (74.9% vs. 25.1%) (Table S1).

**TABLE 1:** Patterns of sensitization to dog allergen components by age and gender in subjects being sensitized to dog dander IgE (e5)^*^(*n* = 313)

Sensitization	All *N =* 313 *n* (%)	≤30 years (*n* = 74)		31–45 years (*n* = 103)		46–60 years (*n* = 96)		61–75 years (*n* = 40)		*p*‐values for differences	
		Men (*n* = 31)	Women (*n* = 43)	Men (*n* = 49)	Women (*n* = 54)	Men (*n* = 47)	Women (*n* = 49)	Men (*n* = 21)	Women (*n* = 19)	Age	Gender
Not sensitized to any measured dog component, *n* (%)	95 (30.4)	9 (29.0)	14 (32.5)	10 (20.4)	18 (33.3)	20 (42.5)	7 (14.3)	11 (52.4)	6 (31.6)	.318	.211
Sensitized to at least one measured dog component, *n* (%)	218 (69.6)	22 (71.0)	29 (67.4)	39 (79.6)	36 (66.7)	27 (57.4)	42 (85.7)	10 (47.6)	13 (68.4)	.318	.211
Sensitized to lipocalins^a^, *n* (%)	174 (55.6)	17 (54.8)	23 (53.5)	33 (67.3)	29 (53.7)	22 (46.8)	34 (69.4)	8 (38.1)	8 (42.1)	.159	.604
Sensitized to Can f 1, *n* (%)	134 (42.8)	14 (45.2)	17 (39.5)	22 (44.9)	23 (42.6)	20 (42.5)	27 (55.1)	6 (28.6)	5 (26.3)	.147	.755
Sensitized to Can f 2, *n* (%)	44 (14.1)	3 (9.6)	7 (16.3)	5 (10.2)	4 (7.4)	11 (23.4)	12 (24.4)	0 (0.0)	2 (10.5)	.005	.557
Sensitized to Can f 4, *n* (%)	76 (24.3)	8 (25.8)	13 (30.2)	11 (22.4)	11 (20.4)	13 (27.6)	15 (30.6)	2 (9.5)	3 (15.8)	.144	.609
Sensitized to Can f 6, *n* (%)	86 (27.5)	8 (25.8)	12 (29.9)	19 (38.8)	14 (25.9)	12 (25.5)	18 (36.7)	3 (14.3)	0 (0.0)	.021	.735
Sensitized to serum albumin^b^, *n* (%)	45 (14.4)	4 (12.9)	5 (11.6)	5 (10.2)	10 (18.5)	8 (17.0)	12 (24.5)	0 (0.0)	1 (5.3)	.043	.167
Sensitized to prostatic kallikrein^c^, *n* (%)	104 (33.2)	12 (38.7)	17 (39.5)	14 (28.6)	15 (27.8)	14 (29.8)	22 (44.9)	3 (14.3)	7 (36.8)	.222	.138
Concomitant sensitization to lipocalins, serum albumin, and prostatic kallikrein, *n* (%)	21 (6.7)	3 (9.6)	5 (11.6)	0 (0.0)	3 (5.5)	3 (6.4)	7 (14.3)	–	–	.025	.075
Poly‐sensitization to dog allergen components^d^, *n* (%)	68 (21.7)	7 (22.6)	9 (20.9)	9 (18.4)	11 (20.4)	13 (27.7)	18 (36.7)	0 (0.0)	1 (5.3)	.002	.387

^*^ Of note, one person can be sensitized to several dog allergen components and thus, the same person can be included in several of the groups. Only first two sensitization groups are mutually exclusive. The percentages in age‐grouped data refer to percentage of subjects in the group having that sensitization pattern as calculated in relation to that age‐ and gender stratified group.

^a^ All subjects sensitized to lipocalin (=sensitization to any of Can f 1, Can f 2, Can f 4, and Can f 6).

^b^ All subjects sensitized to albumin (=sensitization to Can f 3).

^c^ All subjects sensitized to prostatic kallikrein (=sensitized to Can f 5).

^d^ Sensitized to 3 or more of the dog allergen components.

### 3.2 Patterns of sensitization to dog allergen components among those sensitized to dog dander

The most common allergen family was the lipocalins (55.6%), followed by prostatic kallikrein (33.2%) and then serum albumin (14.4%) (Table 1). Among the lipocalins, Can f 1 was the allergen to which most subjects were sensitized (42.8%), while Can f 2 was the allergen to which fewest were sensitized (14.1%) (Table 1). Concomitant sensitization to the three allergen component families was present in 6.7% of participants, while 21.7% were polysensitized (i.e. sensitized to 3 or more allergen components) across the specific allergen components (Table 1). Within each age category, we did not observe any difference in the frequency of sensitization between males and females, but there were age differences in sensitization to Can f 2, Can f 6, concomitant sensitization to all allergen component families and polysensitization: In each of these cases, sensitization was lowest among the oldest age group and highest among those 46–60 years of age (Table 1).

### 3.3 Sensitization patterns by participants' characteristics among those sensitized to at least one dog allergen component

Among those sensitized to at least one dog allergen component (*n* = 218), sensitization to the lipocalins did not differ by any participants' characteristics, except for BMI, in which case the frequency of sensitization was lowest among obese subjects than those with lower BMI (Table 2). Sensitization to serum albumin was the lowest among obese subjects (BMI ≥ 30) and highest among overweight subjects (BMI: 25–29.9); lower in subjects living in densely populated towns than among those living in smaller towns or villages; higher among those who currently own a dog than those without a dog; higher among subjects with asthma than those without asthma (25.6% vs. 6.9%) as well as subjects with both asthma and allergic rhinitis (26.1% vs. 11.9%) (Table 2). Sensitization to prostatic kallikrein did not differ by subjects' characteristics, except for current dog ownership, in which the frequency of sensitization was higher among those who currently own a dog than among those without a dog (65.7% vs. 44.3%) (Table 2).

**TABLE 2:** Background characteristics of the subjects sensitized to at least one dog allergen component in relation to sensitization to dog allergen component families^*^ (*n* = 218)

Background characteristic	Sensitized to any specific dog allergen component *n* = 218 *n* (%)	Sensitized tolipocalins^a^ *n* = 174 (79.8%) *n* (%)	*p*‐value	Sensitized to albumin^b^ *n* = 45 (20.6%) *n* (%)	*p*‐value	Sensitized to prostatic kallikrein *n* = 104 (47.7%) *n* (%)	*p*‐value
Gender							
Males	98 (45.0)	80 (81.6)	.546	17 (17.3)	.277	43 (43.9)	.306
Females	120 (55.0)	94 (78.3)		28 (23.3)		61 (50.8)	
Age, years							
≤30	51 (23.4)	40 (78.4)	.569	9 (17.6)	.073	29 (56.9)	.180
31–45	75 (34.4)	62 (82.7)		15 (20.0)		29 (38.7)	
46–60	69 (31.7)	56 (81.2)		20 (29.0)		36 (52.2)	
61–75	23 (10.6)	16 (69.6)		1 (4.3)		10 (43.5)	
Smoking status							
Non‐smokers	129 (59.4)	99 (76.7)	.396	25 (19.4)	.542	64 (49.6)	.811
Ex‐smokers	54 (24.8)	46 (85.2)		14 (25.9)		24 (44.4)	
Current smokers	34 (15.6)	28 (82.4)		6 (17.6)		16 (47.1)	
Missing information	1 (0.5)	1 (100.0)		0		0	
BMI, kg/m^2^							
<25	83 (38.1)	71 (85.5)	.010	11 (13.3)	.002	35 (42.2)	.111
25–29.9	91 (41.7)	75 (82.4)		29 (31.9)		42 (46.2)	
≥30	44 (20.2)	28 (63.6)		5 (11.4)		27 (61.4)	
Exposure to dust/fumes at workplace							
No	169 (77.5)	133 (78.7)	.360	37 (21.9)	.320	81 (47.9)	.784
Yes	46 (21.1)	39 (84.8)		7 (15.2)		21 (45.7)	
Missing information	3 (1.4)	2 (66.7)		1 (33.3)		2 (66.7)	
Raise on a farm							
No	203 (93.1)	163 (80.3)	.710	42 (20.7)	1.000	96 (47.3)	.855
Yes	12 (5.5)	9 (75.0)		2 (16.7)		6 (50.0)	
Missing information	3 (1.4)	2 (66.7)		1 (33.3)		2 (66.7)	
Degree urbanization							
>10,000 inhabitants	156 (71.6)	125 (80.1)	.856	22 (14.1)	<.001	74 (47.4)	.899
≤10,000 inhabitants	62 (28.4)	49 (79.0)		23 (37.1)		30 (48.4)	
Highest education attained							
Less than high school	21 (9.6)	18 (85.7)	.410	5 (23.8)	.838	12 (57.1)	.533
High school	97 (44.5)	80 (82.5)		21 (21.6)		43 (44.3)	
Tertiary	100 (45.9)	76 (76.0)		19 (19.0)		49 (49.0)	
Family history of allergy or asthma							
No	77 (35.3)	61 (79.2)	.871	21 (27.3)	.074	38 (49.4)	.719
Yes	141 (64.7)	113 (80.1)		24 (17.0)		66 (46.8)	
Currently owns a dog							
No	183 (83.9)	148 (80.9)	.374	29 (15.8)	<.001	81 (44.3)	.020
Yes	35 (16.1)	26 (74.3)		16 (45.7)		23 (65.7)	
Current asthma							
No	58 (26.6)	42 (72.4)	.101	4 (6.9)	.003	30 (51.7)	.475
Yes	160 (73.4)	132 (82.5)		41 (25.6)		74 (46.3)	
Current asthma and allergic rhinitis							
No	84 (38.5)	66 (78.6)	.717	10 (11.9)	.012	39 (46.4)	.765
Yes	134 (61.5)	108 (80.6)		35 (26.1)		65 (48.5)	
Current asthma without allergic rhinitis							
No	192 (88.1)	150 (78.1)	.091	39 (20.3)	.744	95 (49.5)	.154
Yes	26 (11.9)	24 (92.3)		6 (23.1)		9 (34.6)	
Current allergic rhinitis							
No	44 (20.2)	36 (81.8)	.711	6 (13.6)	.199	18 (40.9)	.312
Yes	174 (79.8)	138 (79.3)		39 (22.4)		86 (49.4)	
Current allergic rhinitis without asthma							
No	178 (81.7)	144 (80.9)	.401	41 (23.0)	.066	83 (46.6)	.502
Yes	40 (18.3)	30 (75.0)		4 (10.0)		21 (52.5)	
Current allergic rhinoconjunctivitis							
No	22 (10.1)	17 (77.3)	.774	5 (22.7)	.783	12 (54.5)	.472
Yes	151 (69.3)	122 (80.8)		31 (20.5)		70 (46.4)	
Missing data	45 (20.6)	35 (77.8)		9 (20.0)		22 (48.9)	

^*^ Of note, one person can be sensitized to several dog allergen components and thus the same person can be included in several of the groups. The percentages in component family‐grouped data refer to percentage of subjects in the group having that sensitization pattern as calculated in relation to the group being sensitized to any dog allergen component. The percentages were calculated according to rows.

^a^ All subjects sensitized to lipocalin (=sensitization to any of Can f 1, Can f 2, Can f 4, and Can f 6) were included.

^b^ All subjects sensitized to albumin (=sensitization to Can f 3) were included.

^c^ All subjects sensitized to prostatic kallikrein (=sensitized to Can f 5) were included.

Monosensitization to the lipocalins was present in 42.7% of the participants, while monosensitization to prostatic kallikrein was present in 16.5% of subjects sensitized to at least one dog allergen component (Table 3). Monosensitization to the lipocalins did not differ by participants' characteristics, except for current dog ownership, in which the frequency of monosensitization was higher among those who did not own a dog than among those who owned a dog (Table 3). Monosensitization to prostatic kallikrein was higher among obese subjects than among those having lower BMI. Monosensitization to prostatic kallikrein was higher among subjects without asthma than those without asthma (25.9% vs. 13.1%) (Table 3).

**TABLE 3:** Background characteristics in relation to mono‐ and polysensitization patterns among subjects sensitized to at least one dog allergen component^*^ (*n* = 218)

Background characteristic	Monosensitization to lipocalins^a^ *n* = 93 (42.7%) *n* (%)	*p*‐value	Monosensitization to prostatic kallikrein^b^ *n* = 36 (16.5%) *n* (%)	*p*‐value	Concomitant sensitization to lipocalins, albumin, and prostatic kallikrein^c^ *n* = 21 (9.6%) *n* (%)	*p*‐value	Polysensitization to dog allergen components^d^ *n* = 68 (31.2%) *n* (%)	*p*‐value
Gender								
Males	46 (46.9)	.248	14 (14.3)	.423	6 (6.1)	.112	29 (29.6)	.645
Females	47 (39.2)		22 (18.3)		15 (12.5)		39 (32.5)	
Age, years								
≤30	21 (41.2)	.612	11 (21.6)	.117	8 (15.7)	.027	16 (31.4)	.002
31–45	34 (45.3)		9 (12.0)		3 (4.0)		20 (26.7)	
46–60	26 (37.7)		9 (13.0)		10 (14.5)		31 (44.9)	
61–75	12 (52.2)		7 (30.4)		0 (0.0)		1 (4.3)	
Smoking status								
Non‐smokers	54 (41.9)	.938	25 (19.4)	.240	12 (9.3)	.536	41 (31.8)	.784
Ex‐smokers	24 (44.4)		5 (9.3)		7 (13.0)		18 (33.3)	
Current smokers	14 (41.2)		6 (17.6)		2 (5.9)		9 (26.5)	
BMI, kg/m^2^								
<25	44 (53.0)	.053	11 (13.3)	.009	7 (8.4)	.558	24 (28.9)	.194
25–29.9	33 (36.3)		11 (12.1)		11 (12.1)		34 (37.4)	
≥30	16 (36.4)		14 (31.8)		3 (6.8)		10 (22.7)	
Exposure to dust/fumes at workplace								
No	71 (42.0)	.480	26 (16.7)	.923	18 (10.7)	.577	55 (32.5)	.402
Yes	22 (47.8)		10 (16.1)		3 (6.5)		12 (26.1)	
Raise on a farm								
No	88 (43.3)	.909	32 (15.8)	.418	20 (9.9)	1.000	62 (30.5)	.522
Yes	5 (41.7)		3 (25.0)		1 (8.3)		5 (41.7)	
Degree urbanization								
>10,000 inhabitants	72 (46.2)	.098	26 (11.6)	.962	10 (6.4)	.011	46 (29.5)	.389
≤10,000 inhabitants	21 (33.9)		10 (11.4)		11 (17.7)		22 (35.5)	
Highest education attained								
Less than high school	8 (38.1)	.905	3 (14.3)	.254	4 (19.0)	.246	8 (38.1)	.765
High school	42 (43.3)		12 (12.4)		7 (7.2)		30 (30.9)	
Tertiary	43 (43.0)		21 (21.0)		10 (10.0)		30 (30.0)	
Family history of allergy or asthma								
No	27 (35.1)	.094	15 (19.5)	.383	9 (11.7)	.477	25 (32.5)	.764
Yes	66 (46.8)		21 (14.9)		12 (8.5)		43 (30.5)	
Currently owns a dog								
No	88 (48.1)	<.001	30 (16.4)	.913	14 (7.7)	.053	53 (29.0)	.104
Yes	5 (14.3)		6 (17.1)		7 (20.0)		15 (42.9)	
Current asthma								
No	26 (44.8)	.697	15 (25.9)	.025	2 (3.4)	.062	9 (15.5)	.003
Yes	67 (41.9)		21 (13.1)		19 (11.9)		59 (36.9)	
Current asthma and allergic rhinitis								
No	40 (47.6)	.241	16 (19.0)	.425	5 (6.0)	.145	21 (25.0)	.118
Yes	53 (39.6)		20 (14.9)		16 (11.9)		47 (35.1)	
Current asthma without allergic rhinitis								
No	79 (41.1)	.219	35 (18.2)	.088	18 (9.4)	.723	56 (29.2)	.079
Yes	14 (53.8)		1 (3.8)		3 (11.5)		12 (46.2)	
Current allergic rhinitis								
No	23 (52.3)	.149	7 (15.9)	.904	3 (6.8)	.580	14 (31.8)	.920
Yes	70 (40.2)		29 (16.7)		18 (10.3)		54 (31.0)	
Current allergic rhinitis without asthma								
No	76 (42.7)	.982	27 (15.2)	.259	19 (10.7)	.380	61 (34.3)	.039
Yes	17 (42.5)		9 (22.5)		2 (5.0)		7 (17.5)	
Current allergic rhinoconjunctivitis								
No	9 (40.9)	.804	4 (18.2)	.760	3 (13.6)	.706	9 (40.9)	.360
Yes	66 (43.7)		24 (15.9)		15 (9.9)		47 (31.1)	
Missing data	1 (2.2)		8 (17.8)		3 (6.7)		12 (26.7)	

^*^ Of note, the percentages were calculated according to rows.

^a^ Sensitized only to the lipocalins Can f 1, Can f 2, Can f 4, and Can f 6.

^b^ Sensitized to only prostatic kallikrein Can f 5.

^c^ Concomittant sensitization to lipocalins, albumin, prostatic kallikrein.

^d^ Sensitized to 3 or more of the dog allergen component.

Concomitant sensitization to the three allergen component families did not occur among the oldest subjects but ranged between 4.0% and 15.7% among younger age groups. Concomitant sensitization was lower in subjects living in densely populated towns (>10,000 inhabitants) than among those living in smaller towns (<10,000 inhabitants) or villages and there was a tendency towards lower concomitant sensitization among non‐dog owners and those with asthma (Table 3). Finally, polysensitization to three or more dog allergen components was lowest among the oldest subjects and highest among middle‐aged subjects than younger subjects (Table 3). Furthermore, polysensitization was higher among subjects with asthma than subjects without asthma (36.9% vs. 15.5%) and also higher in subjects who had allergic rhinitis without asthma (34.3% vs. 17.5%) than those without (Table 3).

### 3.4 Overlap of sensitization to molecular dog allergen families among those sensitized to at least one molecular allergen

**FIGURE 1:**
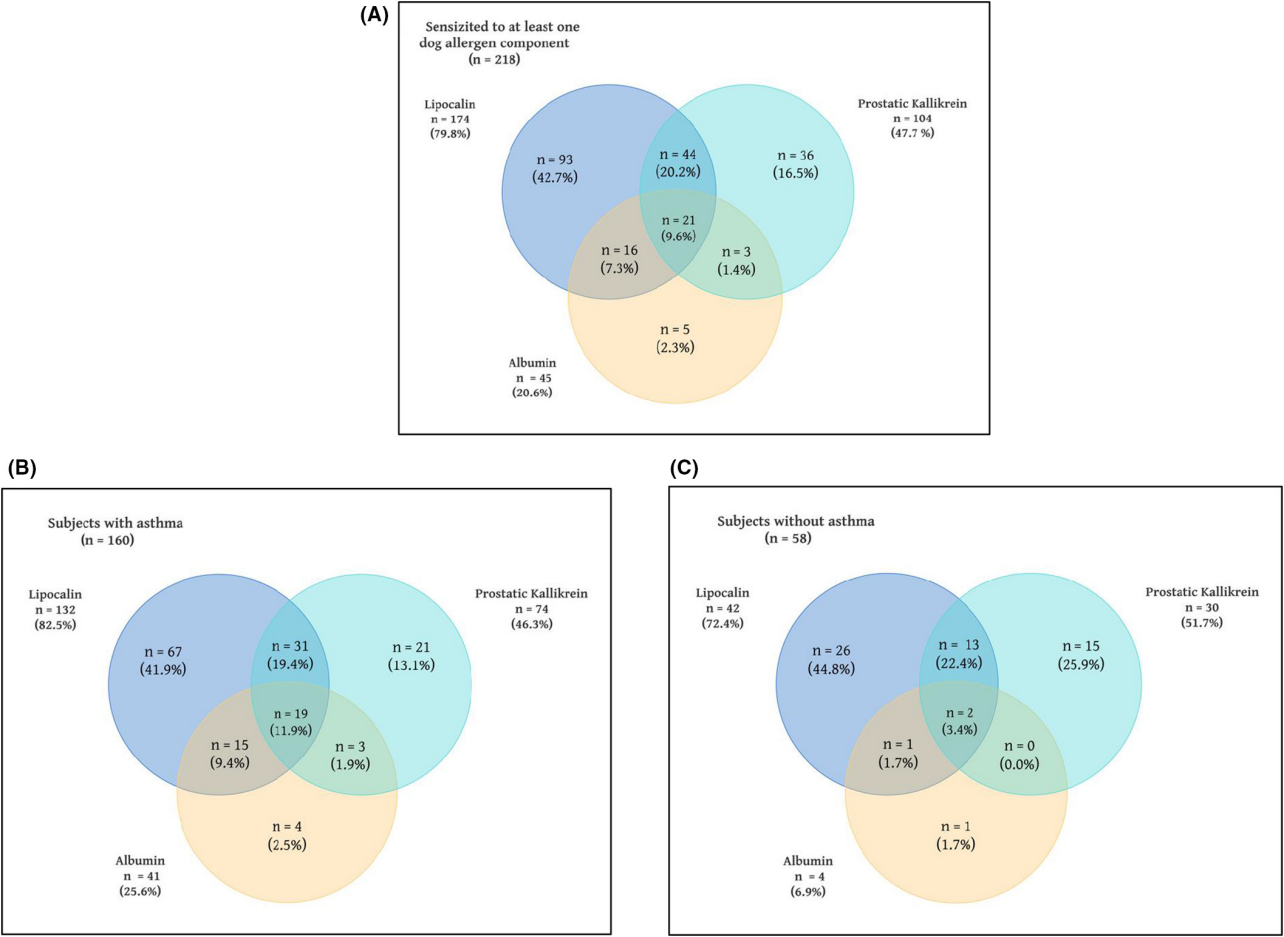
Venn diagram of the sIgE positivity for lipocalins (Can f 1, Can f 2, Can f 4 and Can f 6), albumin (Can f 3), and prostatic kallikrein (Can f 5) among those being found sensitized to at least one dog allergen component (A); among subjects with asthma (B); and without asthma (C). %, percentage of those sensitized to respective allergen components within each group; sIgE, specific immunoglobulin E. *Of note, one person can be sensitized to several dog allergen components and thus, the same person can be included in several of the groups.

**FIGURE 2:**
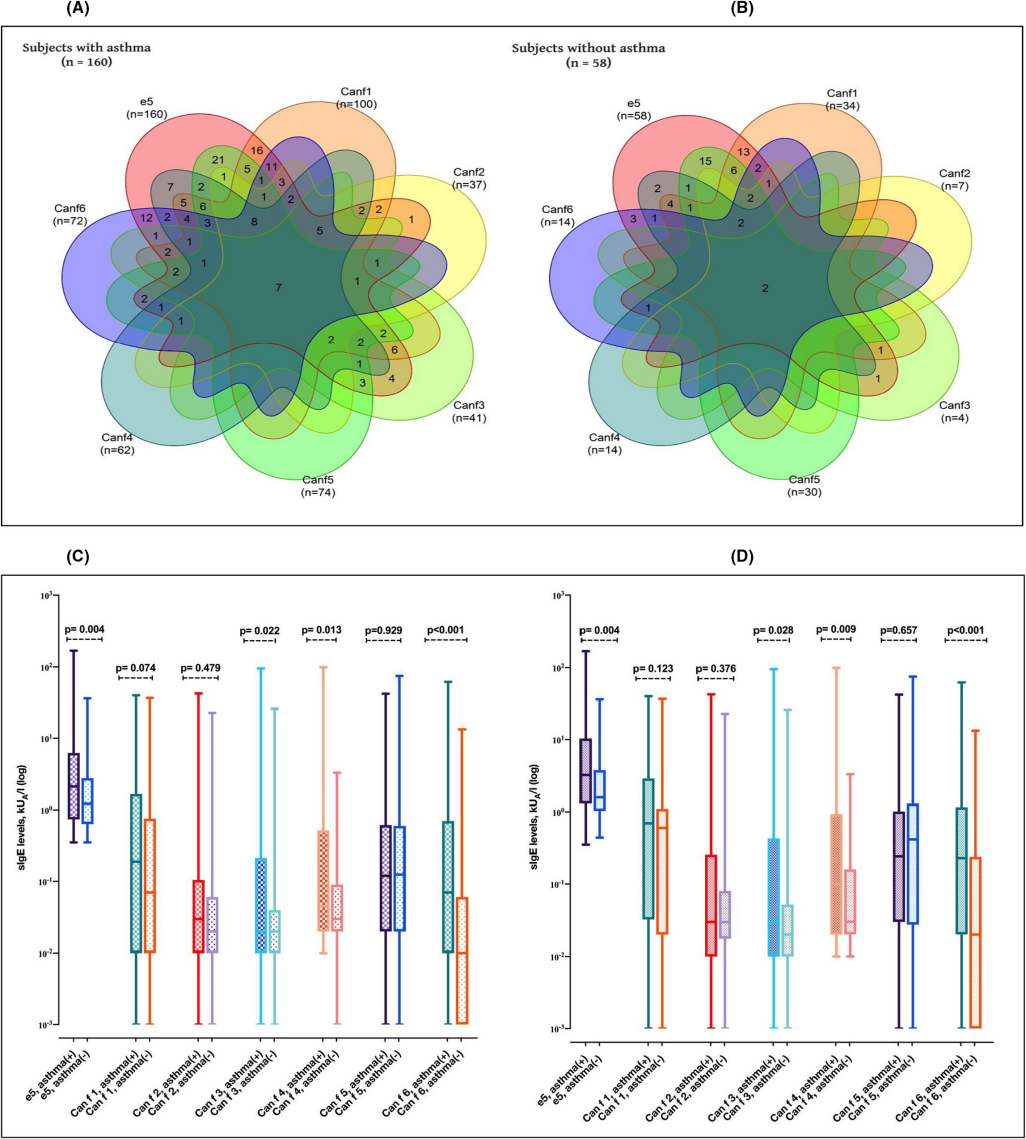
Higher levels of sIgE to dog allergen components a seen more often in subjects with asthma than subjects without asthma. Venn diagram of the sIgE positivity to dog allergen components in subjects with asthma (A) and without asthma (B). Comparison of median sIgE levels to each dog allergen component by the presence of asthma vs non‐asthma (asthma *n* = 221, non‐asthma *n* = 92) in all study group (C). Comparison of median sIgE levels to each dog allergen component by the presence of asthma vs non‐asthma (asthma *n* = 160, non‐asthma *n* = 58) among subjects sensitized to at least one dog allergen component (D). e5, dog dander immunoglobulin E; sIgE, specific immunoglobulin E. In (C,D) data are presented as median, and whiskers indicate the minimum and maximum values. *Of note, one person can be sensitized to several dog allergen components and thus, the same person can be included in several of the groups. Whiskers indicate the minimum and maximum values.

Among subjects who were sensitized to at least one component (*n* = 218), 9.6% were simultaneously sensitized to all three protein families, but it was 11.9% and 3.4% between subjects with and without asthma, respectively (Figure 1). The most common overlapping sensitization pattern was co‐sensitization to lipocalins and prostatic kallikrein (29.8%), while the least common was co‐sensitization to prostatic kallikrein and serum albumin (11.0%) (Figure 1A). The overlaps between the lipocalins and prostatic kallikrein were similar between those with and without asthma (31.3% vs. 25.9%). On the other hand, the frequency of overlap between the lipocalins and serum albumin was higher in individuals with asthma than those without asthma (21.3% vs. 5.2%) (Figure 1B,C). Similarly, individuals with asthma had higher rates of overlapping sensitization to prostatic kallikrein and serum albumin than those without asthma (13.8% vs. 3.4%) (Figure 1B,C). A more detailed characterization of the overlapping sensitization patterns for single dog allergen components regarding the presence of asthma is shown in Figure 2, indicating that any overlapping sensitization to specific allergen components was more common in those with asthma than those without asthma (Figure 2A,B).

**FIGURE 3:**
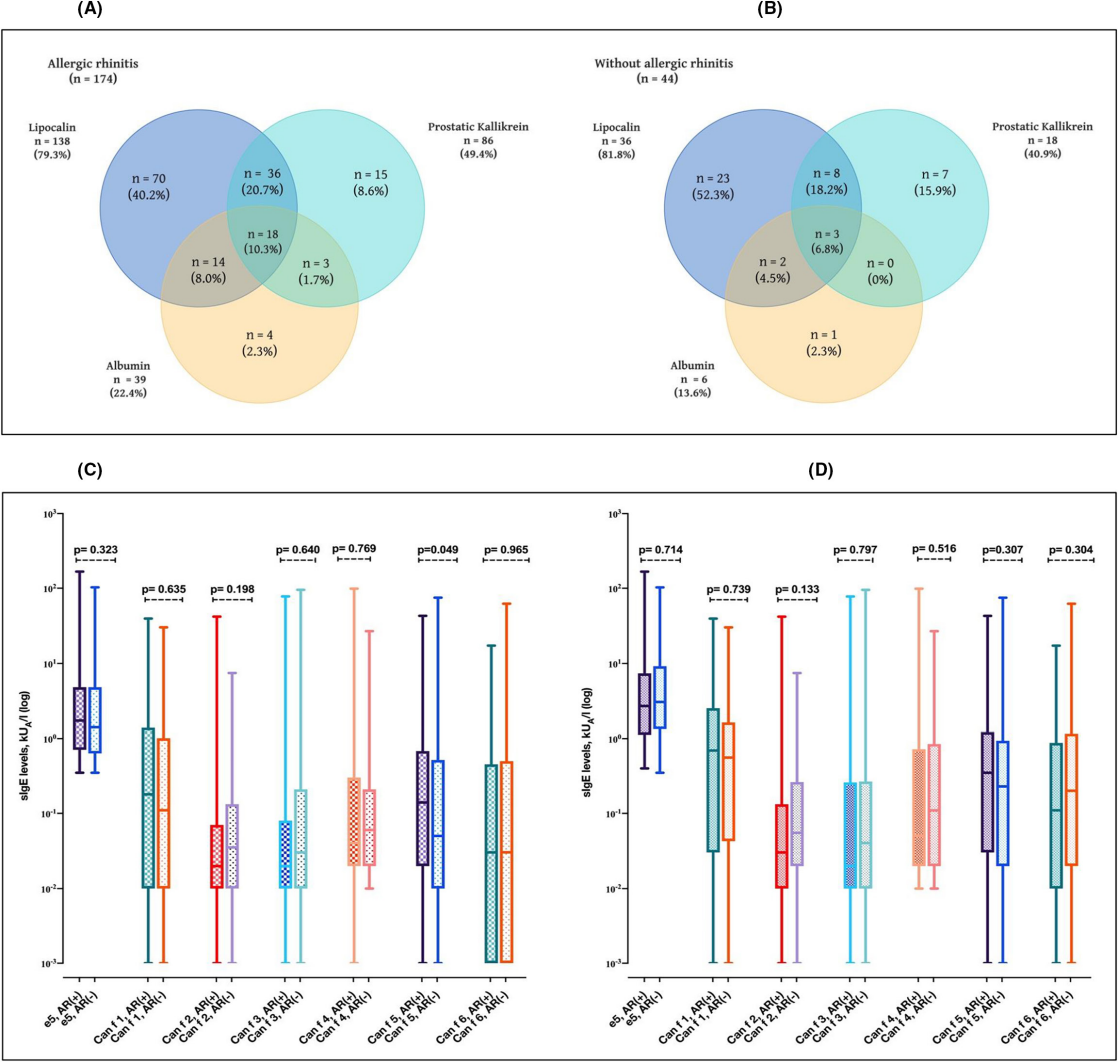
Higher levels of Can f 5 are seen more often in subjects with allergic rhinitis than those without allergic rhinitis. Venn diagram of the sIgE positivity to lipocalin, albumin and prostatic kallikrein in subjects with allergic rhinitis (A), without allergic rhinitis (B). Comparison of median sIgE levels to each dog allergen component by the presence of allergic rhinitis (AR) vs without AR (AR *n* = 245, no AR *n* = 68) in all study group (C). Comparison of median sIgE levels to each dog allergen component by the presence of allergic rhinitis vs without AR (AR *n* = 174, no AR *n* = 44) among subjects sensitized to at least one dog allergen component (D). %, percentage of those sensitized to respective allergen components within each group; e5, dog dander immunoglobulin E; sIgE, specific immunoglobulin E. In (C,D) data are presented as median, and whiskers indicate the minimum and maximum values. *Of note, one person can be sensitized to several dog allergen components. and thus the same person can be included in several of the groups.

The frequencies of overlapping sensitization were higher in subjects with than those without allergic rhinitis in every case (Figure 3A,B). Concomitant sensitization to all three protein families was 10.3% in subjects with allergic rhinitis, while it was 6.8% in subjects without allergic rhinitis (Figure 3A,B). Furthermore, subjects who had allergic rhinitis without asthma yielded lower rates of concomitant sensitization than individuals with allergic rhinitis and asthma (5.0% vs. 11.9%) (Figure S2).

**FIGURE 4:**
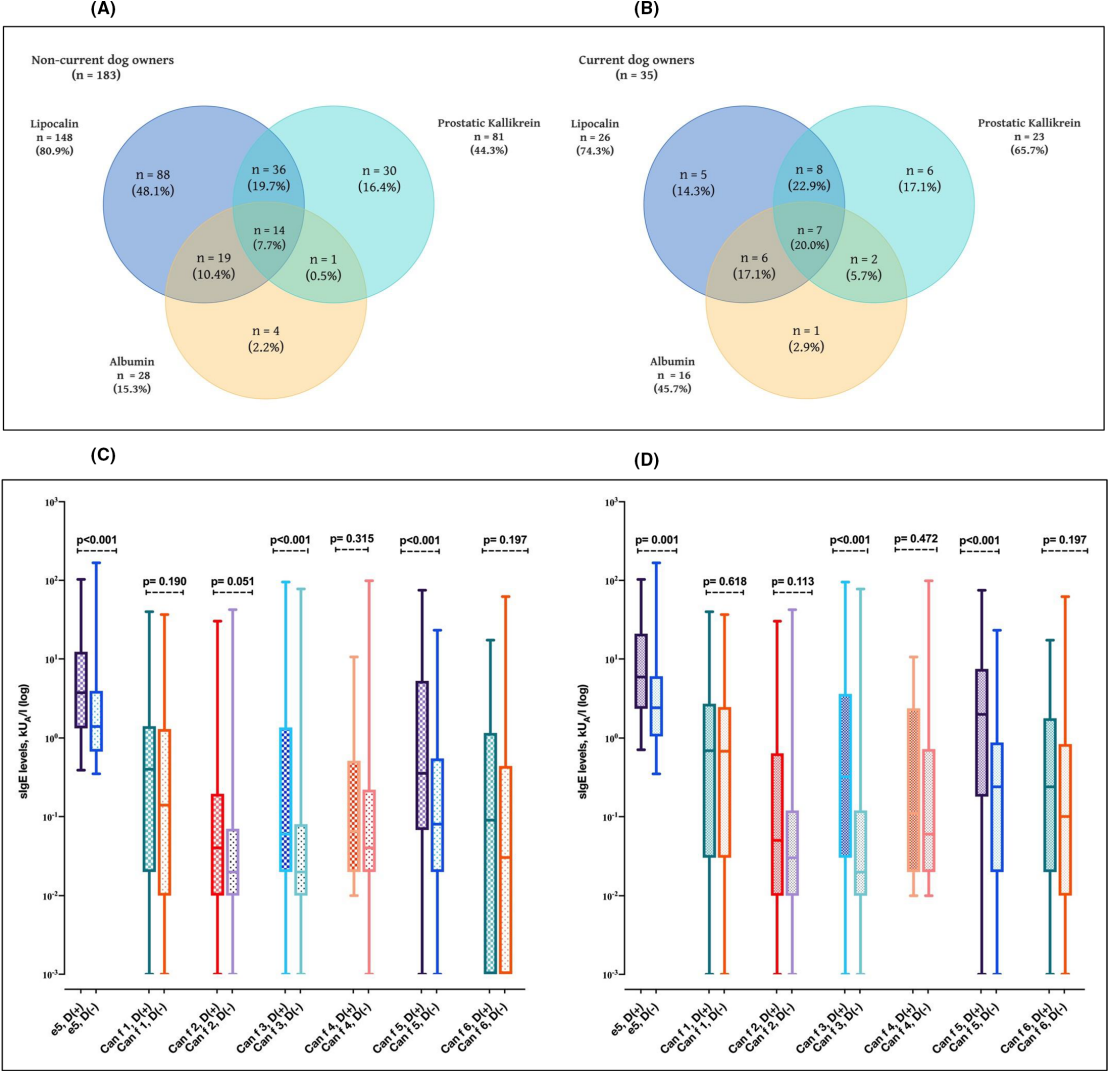
Current dog owners show increased levels of sIgE to Can f 3 and Can f 5. Venn diagram of the sIgE positivity for lipocalins, albumin and prostatic kallikrein among non‐current dog owners (A); among current dog owners (B). Comparison of median sIgE levels to each dog allergen component by current dog ownership (current dog owners *n* = 46, non‐current dog owners, *n* = 267) in all study group (C). Comparison of median sIgE levels to each dog allergen component by current dog ownership (current dog owners *n* = 35, non‐current dog owners, *n* = 183) among subjects sensitized to at least one dog allergen component (D). %, percentage of those sensitized to respective allergen components within each group. D, current dog ownership; e5, dog dander immunoglobulin E; sIgE, specific immunoglobulin E. In (C,D) data are presented as median, and whiskers indicate the minimum and maximum values. *Of note, one person can be sensitized to several dog allergen components and thus, the same person can be included in several of the groups.

The frequencies of overlapping patterns were also higher in subjects with a dog than in those without a dog (Figure 4A,B). Current dog owners had higher rates of concomitant sensitization compared to non‐current dog owners (20.0% vs. 7.7%, respectively) (Figure 4A,B).

While the overlapping sensitizations were similar between females and males (Figure S3), the frequencies of overlapping between lipocalin and serum albumin were higher in non‐obese (19.5%) than in obese (6.8%) subjects (Figure S4). The frequencies of overlap were highest in current smokers, followed by ex‐smokers and non‐smokers (Figure S5).

### 3.5 Median IgE levels of dog dander IgE and molecular dog allergens by sensitization patterns

Analyses of IgE levels to dog dander IgE and dog molecular allergens were implemented in the whole sample (*n* = 313) and then repeated within subjects who were sensitized to at least one dog allergen component (*n* = 218). IgE levels to dog dander were significantly higher in subjects with asthma than those without asthma (1.22 vs. 2.13 kU_A_/L, *p* = .004) but not between those with and without allergic rhinitis (1.41 vs. 1.72 kU_A_/L, *p* = .323).

**FIGURE 5:**
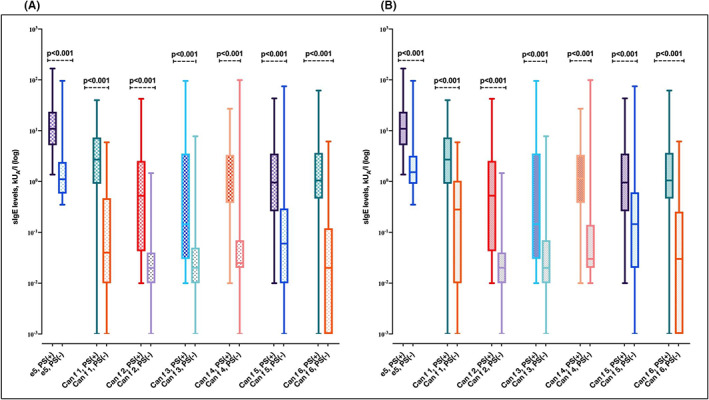
Polysensitized subjects display increased levels of sIgE to each molecular allergen component compared to non‐polysensitized subjects. Comparison of median sIgE levels to dog allergen components by polysensitization (*n* = 68) vs no polysensitization (*n* = 245) in all study group (A). Comparison of median sIgE levels to dog allergen components by polysensitization (*n* = 68) vs no polysensitization (*n* = 150) among subjects sensitized to at least one dog allergen component (B). e5, dog dander immunoglobulin E; PS, polysensitization; sIgE, specific immunoglobulin E. Data are presented as median, and the minimum and maximum values.

The median IgE levels of Can f 3, Can f 4 and Can f 6 were higher among those with than those without asthma and showed a similar pattern in both groups (Figure 2C,D). Among the whole study group, only median Can f 5 levels differed between those with and those without allergic rhinitis (Figure 3C). However, this finding did not remain significant when repeated in subjects sensitized to at least one dog allergen component (Figure 3B). Can f 3, Can f 5 and dog dander IgE levels were significantly higher in current than in non‐current dog owners (Figure 4C,D). The median IgE levels of all the allergen components were statistically significantly higher in polysensitized than non‐polysensitized individuals (Figure 5).

Among the lipocalins, the median IgE levels to the specific allergen components were higher in Can f 1 and Can f 6 monosensitized individuals than in those not monosensitized to lipocalins (Figure S6A). Median IgE levels were higher in those monosensitized than in those not monosensitized to prostatic kallikrein. (Figure S7). The median IgE levels of each component were higher in those with than in those without concomitant sensitization to the three allergen component families (Figure S8).

## 4 DISCUSSION

This study provides a detailed characterization of the sensitization patterns to dog molecular allergens in a population‐based adult sample. Lipocalins were the most common sensitized allergen family, while sensitization to serum albumin was the least common. Among the specific molecular allergens, IgE positivity to Can f 1 was the most common, followed by Can f 5. Sensitization patterns and sIgE levels varied regarding the presence of asthma, allergic rhinitis and current dog ownership. Finally, the median IgE levels to each specific molecular allergen were usually higher among individuals with concomitant sensitization and those with polysensitization than those not having these sensitization patterns.

In the current literature, there is a paucity of data about dog allergen components in adults as most studies have focused on children. Can f 1 and Can f 5 are considered to be the major allergens for dog allergy,^19^, ^27^ but Can f 1 is more common and may be as high as 50%–90% within dog sensitized subjects.^13^ Our findings were mainly in line with the previous literature, indicating that Can f 1 may be the most common molecular dog allergen, followed by Can f 5 both in adults and children.^16^, ^28^ However, some previous studies have also found that sensitization to Can f 5 was higher than Can f 1.^29^, ^30^ That difference might stem from the source of the population with regard to age, environmental, and geographical factors, as well as different diagnostic techniques.

Generally, major allergens are likely to be more related to clinical outcomes than minor allergens and these were previously shown to be risk factors for asthma.^24^, ^30^, ^31^ Sensitization to Can f 1 and Can f 5 were associated with asthma in two previous paediatric studies.^30^, ^31^ We found that sIgE levels to Can f 5 were higher in subjects with than those without allergic rhinitis, while it did not differ between subjects with and without asthma. On the other hand, monosensitization to Can f 5 was more common in those without than in those with asthma according to our data. Hence, monosensitization to Can f 5 could be also a separate sensitization pattern among subjects with dog allergy. Käck and colleagues (2018) suggested that monosensitization to Can f 5 was inversely related to the positive nasal provocation test, which is in parallel with our findings.^16^ Monosensitization to Can f 5 could be a candidate inverse marker of allergic symptoms and should be separately investigated.

Can f 5 is excreted from the prostatic tissue, so it only exists in male dogs.^27^ Schools and colleagues (2019) suggested that children monosensitized to Can f 5 showed different responses to male and female dogs.^32^ Therefore, the source of extract, with regard to the sex of the dog, might affect the results. A previous study revealed that sensitization to Can f 5 was significantly higher in owners of a male dog than in owners of a female dog, while there was no association with the owner's gender.^33^ We did not find any significant differences in sensitization to Can f 5 between male and female participants. Unfortunately, we did not collect information on the sex of the dog in our study. Hence, the role of the sex of a dog still remains a future research question.

Among minor allergens (Can f 2, Can f 3, Can f 4 and Can f 6), sensitization to Can f 6 was the most common, similar to previous findings.^20^ IgE values of Can f 3, Can f 4 and Can f 6 were significantly higher in those with than in those without asthma. A recent study showed that subjects with troublesome asthma had higher IgE values against Can f 2, Can f 4 and Can f 6.^34^ Although, these molecules are mainly considered as minor allergens, given their patterns of sensitization in the current study, accurate diagnosis, as well as their clinical relevance on asthma severity and prognosis in adults, requires further investigations. Importantly, Can f 2 and Can f 6 are present in very low amounts in SPT solutions.^12^ Addition to that, Can f 3 also shows considerable variation between 9% and 98% in different SPT extracts.^12^ Therefore, molecular techniques could provide standardized and accurate identification of minor allergens compared to SPT.

We also identified a subgroup of individuals (30%) who was not sensitized to any of the dog molecular allergens, despite the fact that they were sensitized to dog dander IgE. This is in accordance with other studies on dog allergy, as well as other allergies.^16^, ^33^, ^35^ Käck and colleagues (2018) identified a similar non‐sensitized group that composed 10% of the dog sensitized subjects.^16^ Hemmer et al^33^ had a mixed age group subjects and observed that 25% were positive on dog extract but negative on dog components. The difference in proportion between both studies might arise from the different cut‐off values of sIgE levels as well as the study population used in the studies. Käck and colleagues used sIgE positivity of ≥0.10 kUA/L in a child sample, while we used 0.35 kUA/L as the cut‐off value in an adult population. A previous study suggested that the cut‐off value of 0.20 kU/L could have a better sensitivity with lower specificity compared with 0.35 kU/L to predict symptoms arising from dog exposure.^36^

We found that individuals with asthma have higher IgE levels to dog dander than those without asthma, while subjects with and without allergic rhinitis did not differ. It has been suggested that the application of the IgE cut‐off values should be separately investigated in relation to clinical outcomes for each allergen group, instead of using a standard cut‐off value.^37^ Addition to that, Letran et al^38^ suggested that a cut‐off value of 2.18 kU/L could better predict the component negative subgroup in house dust mite allergens.

Nevertheless, these results might also indicate a need for the identification of new molecular allergens to which current non‐sensitized individuals might be sensitized.^16^, ^24^ Two new dog allergen molecules, Can f 7 and Can f 8, have been recently identified and added to the WHO/IUIS Allergen Nomenclature database, after the analyses in our study and the study by Käck and colleagues were performed.^18^, ^39^, ^40^ Another explanation could be the presence of α‐Gal in cat and dog dander extracts.^41^ Kiewiet et al,^42^ in a recent study of patients with α‐Gal syndrome, found a high frequency of sensitization to both dog and cat extracts but a low frequency of genuine cat and dog sensitization using CRD. Considering the challenges of diagnosis and treatment of dog allergy, defining new allergen molecules still needs future research, perhaps those unsensitized to any of the current molecules may be positive to new allergens.

The whole extract positive, but component negative group might also stem from the lower analytical sensitivity of the molecular assay compared to the extract‐based assay.^24^ The analysis of the molecular allergens was undertaken using the singleplex testing based on ImmunoCAP as against the multiplex testing based on microarray technology (ISAC), which has been used by most previous studies. One of the differences between the singleplex and multiplex systems is that the degree of resolution differs between the two, favouring singleplex testing.^43^ In addition, given that the chip‐based technology of the multiplex system is less quantitative, the singleplex IgE antibody assays remain superior for routine diagnostic allergy testing.^43^, ^44^ IMMULITE and HyTEC88 are the other commonly used singleplex tests.^24^, ^45^ Although the inter‐assay correlation was significant between ImmunoCAP and IMMULITE, the assay results are not interchangeable.^45^ Since sIgE values of these three assays showed difference at any particular specificity, methodological differences also should be taken into account while implementing the results.^24^

Total and specific IgE levels to whole extracts were shown to be affected by age, gender and smoking status in previous studies.^46^ Our results suggest that sensitization was less common in older adults than in younger ones. Perzanowski and colleagues (2016) showed that children diagnosed with asthma after the age of 12 tended to be less sensitized to mammalian dog dander and had lower IgE levels than children diagnosed before the age of 12.^31^ Considering the increasing prevalence of dog ownership over time, a cohort effect could contribute to this difference.^47^ Therefore, age might also play a key role in the sensitization profiles of dog allergen components.

Polysensitization is an important consideration in the course of asthma.^48^ Molecular diagnostic techniques might help to identify polysensitization and the sensitization patterns of a specific allergy. In mite and cockroach allergy, increased IgE positivity and higher sIgE levels of molecular allergens were associated with higher presence of asthma and rhinitis.^35^, ^49^ Similarly, in dog allergy, a recent study showed that the number of IgE positivity to dog molecular allergens positively correlated with allergic symptoms.^33^ Polysensitized subjects had higher specific IgE levels, and they were more likely to be current dog owners and live in small towns. These results might indirectly indicate that polysensitized subjects had higher exposure to dog allergens. Considering that both higher IgE levels and polysensitization are risk factors for asthma severity, polysensitization and IgE levels of dog allergen components need further exploration in terms of clinical outcomes in adults, given the current paucity of data on this aspect.^6^, ^34^, ^48^, ^50^

The West Sweden Asthma Study is a population‐representative sample of adults; thus, our results have direct generalizability to the source population. However, given the varying distribution and exposure to dogs across the world, the results may not be applicable to other populations beyond the study´s immediate target population. At the time of publishing our previous paper that characterized the sensitization patterns to furry animal allergen components, the dog allergen components, Can f 4 and Can f 6, were still to be analysed in our cohort; thus the six dog allergen components (Can f 1 to Can f 6) included in the current study represent a comprehensive list of the most clinically important dog allergen components now commercially available.^51^

The increase in pet ownership also should be taken into account since the sample collection of the WSAS 1 was performed between 2009 and 2012.^26^ The number of registered dogs has increased approximately by 39%, which corresponds to one million dogs in Sweden during the last decade.^52^ Allergic sensitization to dogs increased in both adults (1994–2009) and children (1996–2006) in Sweden.^8^, ^53^ On the other hand, the effect of pet ownership on the development of allergic diseases still remains inconsistent. Recently, a meta‐analysis from EU Child Cohort Network showed that pet ownership during early life was not associated with allergic sensitization to dogs.^54^ According to Liccardi and colleagues, indirect allergen exposure should be investigated, in addition to pet ownership, to evaluate the real exposure to dog allergens in a more accurate way.^55^, ^56^ Nonetheless, the increase in dog ownership in parallel with an increase in dog allergy is a global phenomenon, contributing largely to increased airborne allergen sensitization, particularly among the population of the Northern Hemisphere. On this note, a detailed description of dog molecular allergens in a population setting of adults, the first of its kind, provides important information to fill the knowledge gap on the topic.

Defining the sensitization profiles and elucidating their impact on asthma and its clinical impact, particularly in adults, may help clinicians to determine disease prognosis in daily practice. Since the accuracy of SPT is affected by the unstandardized extracts and shows a variance based on the source of extract, the usage of the CRD as a diagnostic tool has become increasingly important in daily practice.^5^ In addition, CRD could guide immunotherapy by identifying sensitization profiles on an individual basis as well as differentiating genuine sensitization from cross‐reactivity.^57^, ^58^ The content and variance of the allergen components in raw materials could be one of the underlying reasons behind the limited effectiveness of immunotherapy, CRD could also improve the therapeutic effectiveness of the dog allergen‐specific immunotherapy by providing the appropriate quantification of allergen components.^4^, ^57^, ^58^, ^59^

Our data show that sensitization to molecular dog allergens presents a complex pattern in adults. The use of CRD may help to improve the accuracy in the diagnosis of dog allergy and provide guidance for disease outcomes and treatment options. There is a need to understand the potential impact of these sensitization patterns on clinical outcomes and the course and severity of asthma in adults.

## AUTHOR CONTRIBUTIONS

SSÖE, HK, LE, BL, JL and BN designed the study. RB, LE and BN are responsible for the study database. LE, MR, BL, CM, JL and BN participated in data collection. SSÖE, HK and BN analysed the data and drafted the manuscript. All authors participated in data interpretation and approved the final manuscript submitted.

## FUNDING INFORMATION

The VBG Group Herman Krefting Foundation for Asthma and Allergy Research, Sweden, is gratefully acknowledged for funding the study. Additional funding was received from Sasakawa Scandinavia Foundation, Japan, the Swedish Research Council, the Swedish Heart‐Lung Foundation, the Swedish Asthma and Allergy Foundation, Thermofisher Scientific and ALF agreement (Västra Götaland) (Grants from the Swedish state under the agreement between the Swedish Government and the county councils).

## CONFLICT OF INTEREST

M.P. Borres is employed by Thermo Fisher Scientific (Uppsala, Sweden). B. Lundbäck and J. Lötvall have received material from Thermo Fisher to perform the IgE analyses for this work. The rest of the authors declare that they have no relevant conflicts of interest related to this work.

## Supporting information


Figure S1‐S8

Table S1‐S2

## Data Availability

The data that support the findings of this study are available on request from the corresponding author. The data are not publicly available due to privacy or ethical restrictions.
