# Supplementary material for: Sensitization to molecular dog allergens in an adult population: Results from the West Sweden Asthma Study
Source: Clin Exp Allergy. 2022 Sep 1;53(1):88–104. doi: 10.1111/cea.14216 (PMC10087160; doi:10.1111/cea.14216)
Supplement: Supplementary file 1 — Figure S1‐S8 [file CEA-53-88-s002.docx]

***SUPPORTING INFORMATION***

Invited to questionnaire survey

30 000

Not traced, unable to participate or disease

782

Final sample contacted for

questionnaire survey

29 218

Non-responders to questionnaire survey

11 131

Responders to questionnaire survey

18 087

Random selected sample

2 000

Asthma sample

1 524

Non-participants from random sample

828

Non-participants from asthma sample

690

Asthma sample

834

Random sample

with asthma

130

Random sample

without asthma

1 042

Participants in

clinical examinations

2 006

No SPT or IgE measurement

134

SPT/IgE measurement

1872

Sensitized to dog dander

(Specific IgE positivity (e5))

313

**Figure S1.** Flowchart of the study population


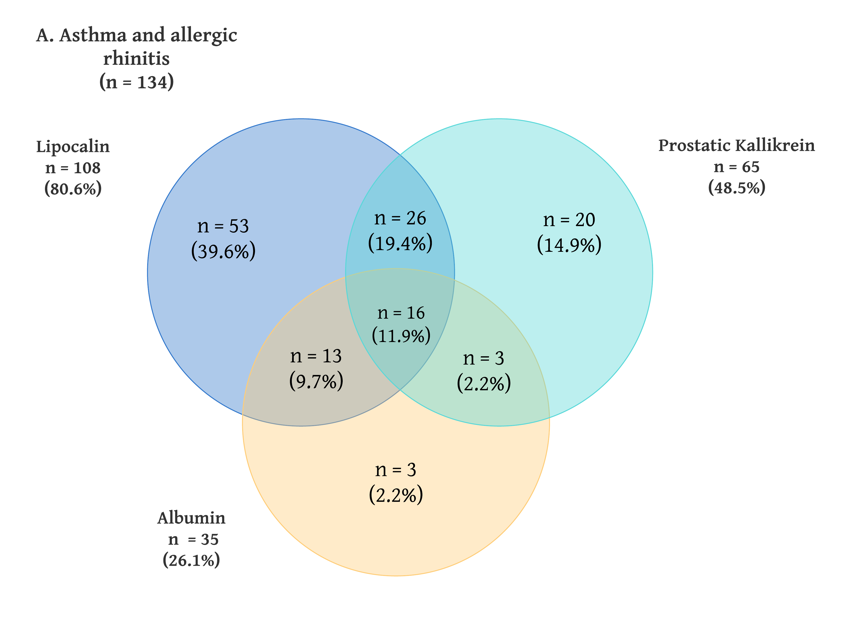

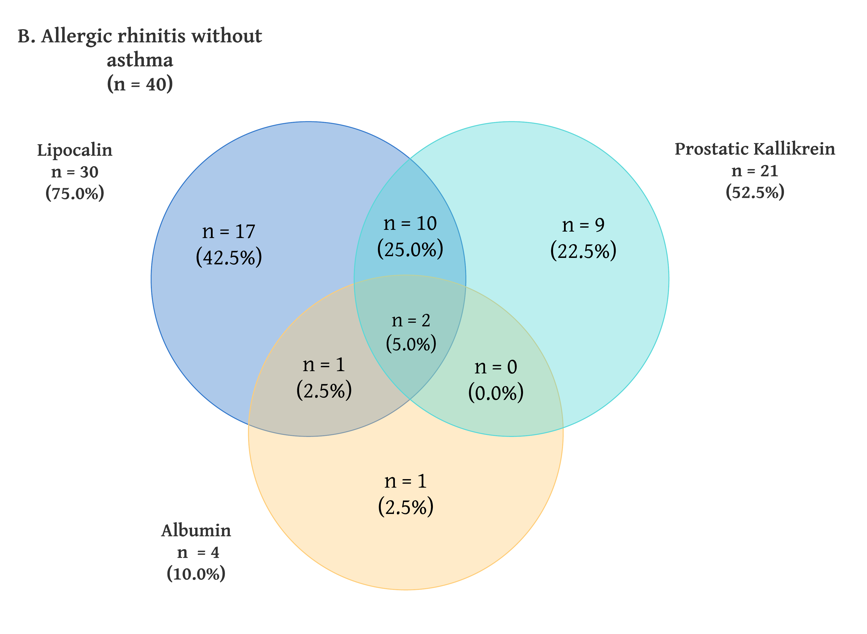


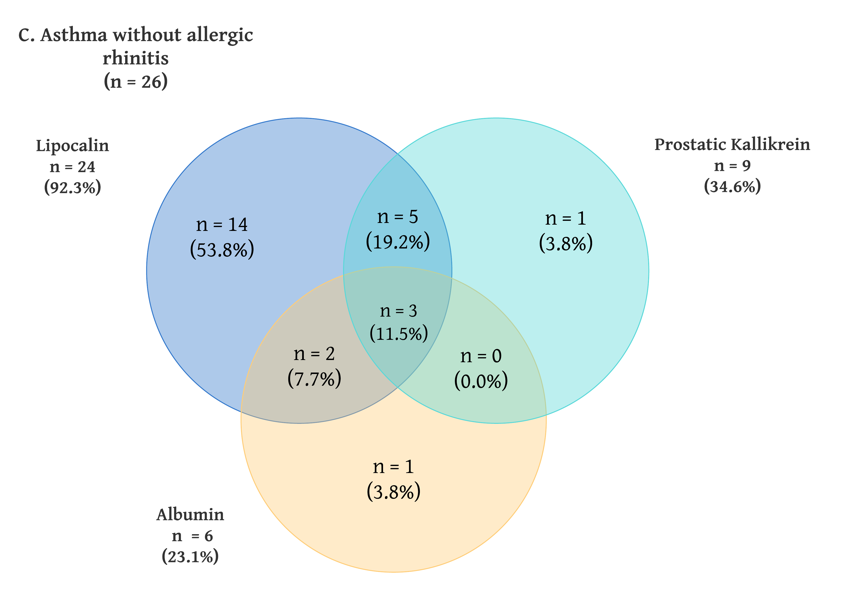

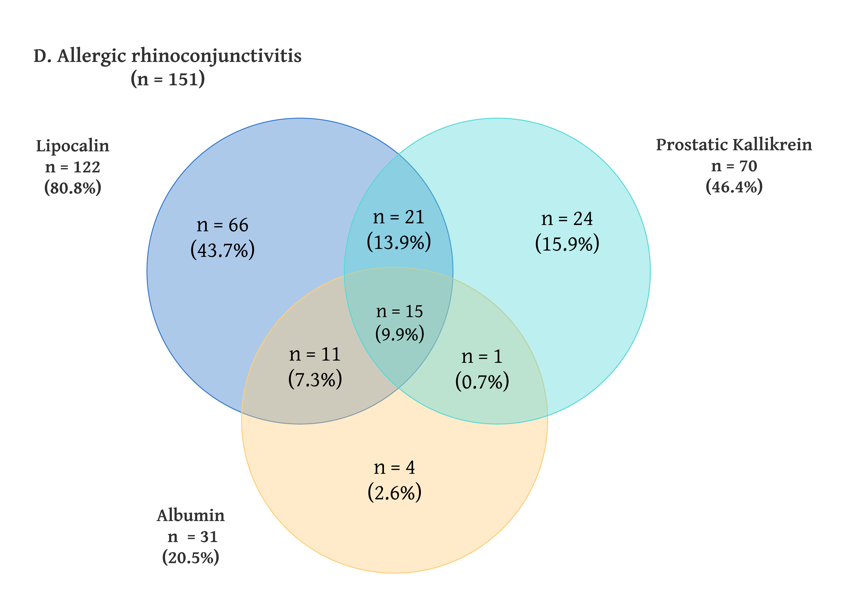


**Figure S2.** Venn diagram of the sIgE positivity to lipocalin, albumin, and prostatic kallikrein in subjects with asthma and allergic rhinitis **(Fig S2A)**, allergic rhinitis without asthma **(Fig S2B**), asthma without allergic rhinitis **(Fig S2C**), and allergic rhinoconjunctivitis **(Fig S2D)** among those being found sensitized to at least one dog allergen component. % = Percentage of those sensitized to respective allergen component within each group.

*Of note, one person can be sensitized to several dog allergen components and thus, the same person can be included in several of the groups.

**
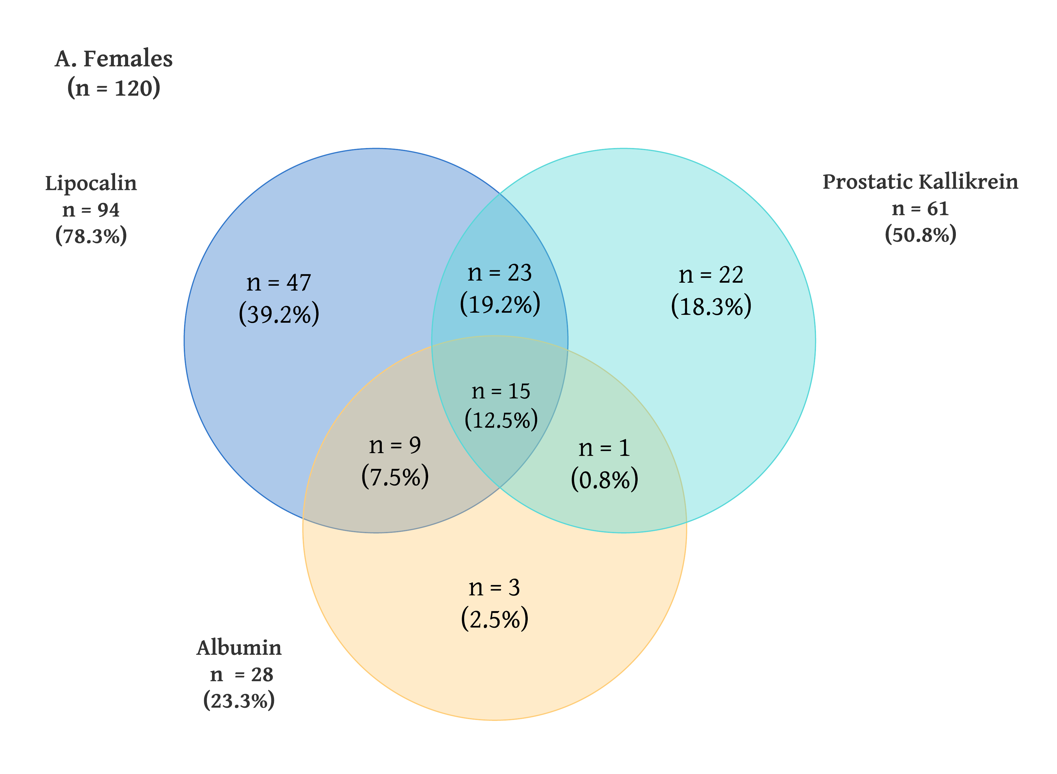

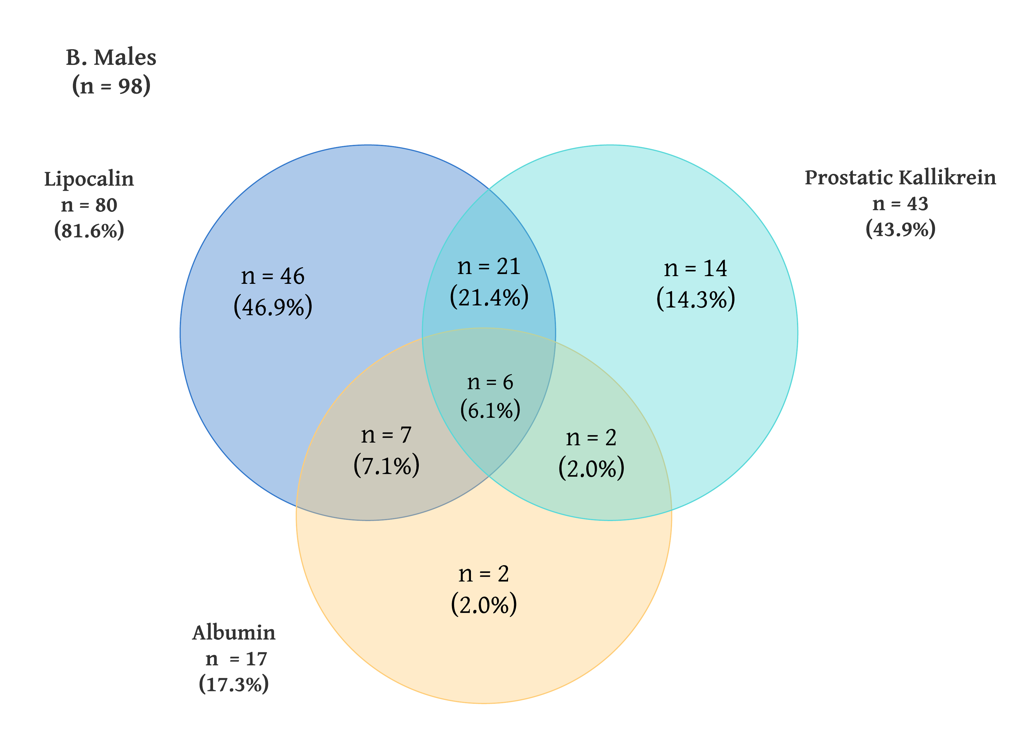
**

**Figure S3.** Venn diagram of the sIgE positivity for lipocalin, albumin and prostatic kallikrein in female **(Fig S3A)** and male participants **(Fig S3B**) among those being found sensitized to at least one dog allergen component. % = Percentage of those sensitized to respective allergen component within each group.

*Of note, one person can be sensitized to several dog allergen components and thus, the same person can be included in several of the groups.

**
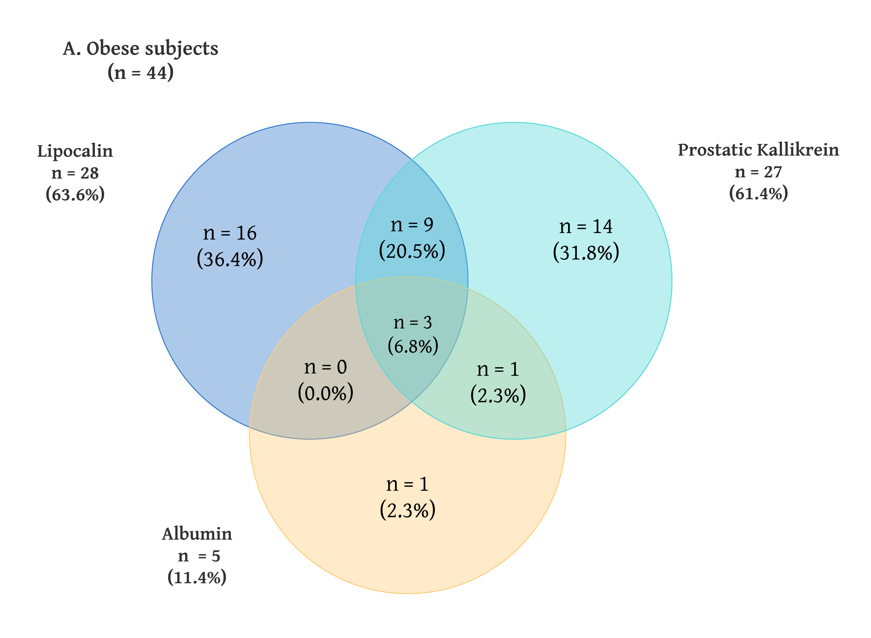

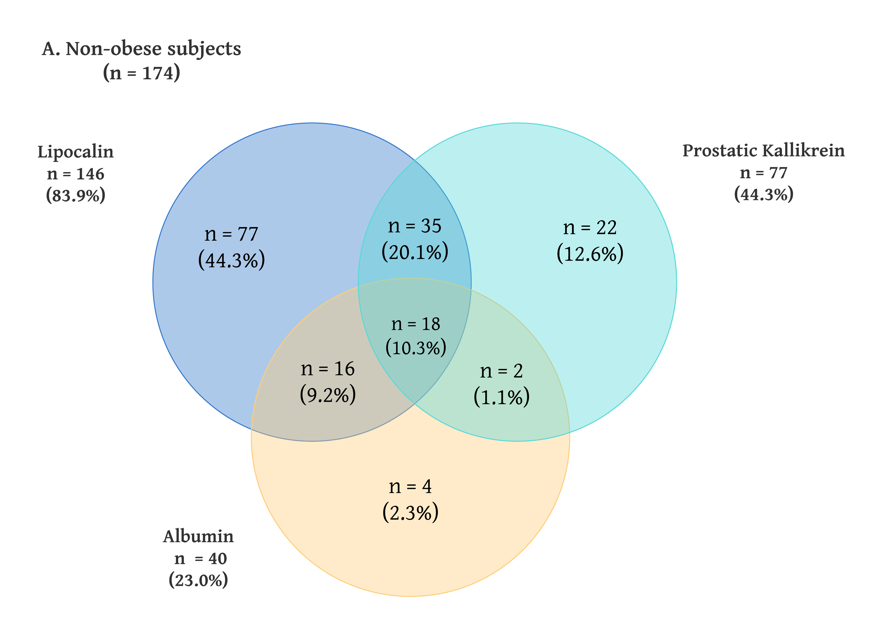
**

**Figure S4.** Venn diagram of the sIgE positivity for lipocalin, albumin and prostatic kallikrein in obese (**Fig S4A**) and non-obese (**Fig S4B**) participants among those being found sensitized to at least one dog allergen component. % = Percentage of those sensitized to respective allergen component within each group.

*Of note, one person can be sensitized to several dog allergen components and thus, the same person can be included in several of the groups.

**
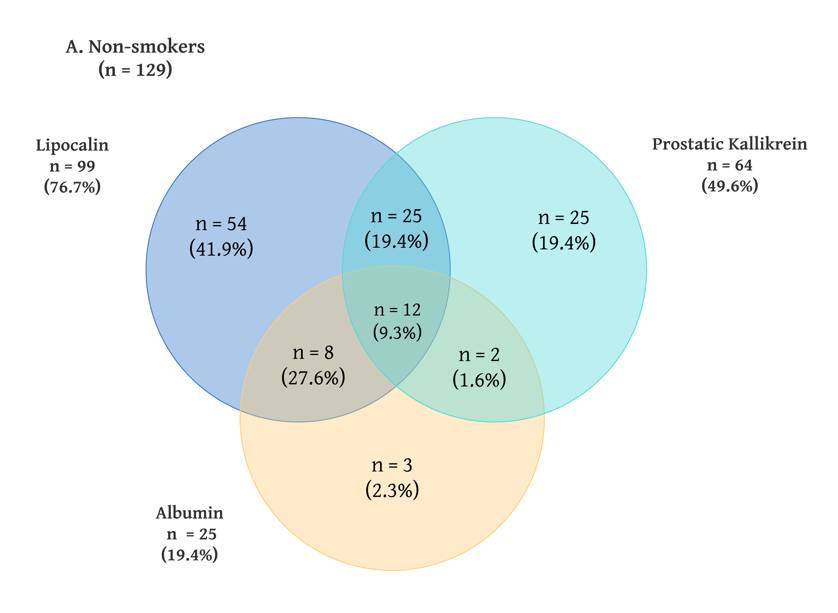

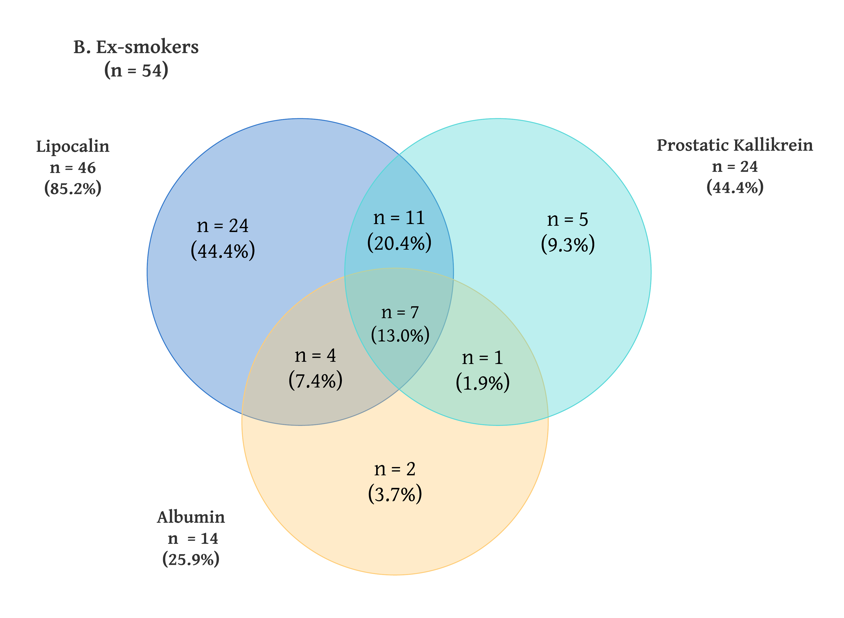
**

**
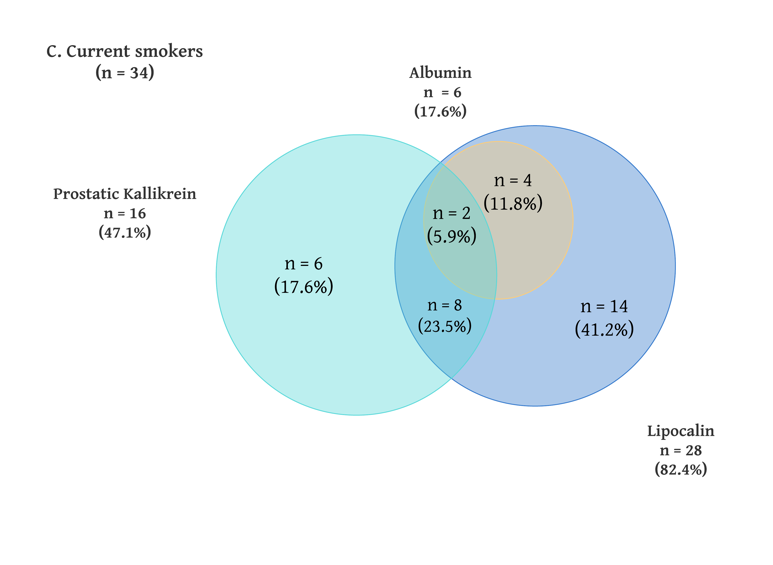
**

**Figure S5.** Venn diagram of the sIgE positivity for lipocalin, albumin and prostatic kallikrein in non-smokers (**Fig S5A),** ex-smokers (**Fig S5B)**, and current smokers (**Fig S5C)** among those being found sensitized to at least one dog allergen component. % = Percentage of those sensitized to respective allergen component within each group.

*Of note, one person can be sensitized to several dog allergen components and thus, the same person can be included in several of the groups.

**A B**

**
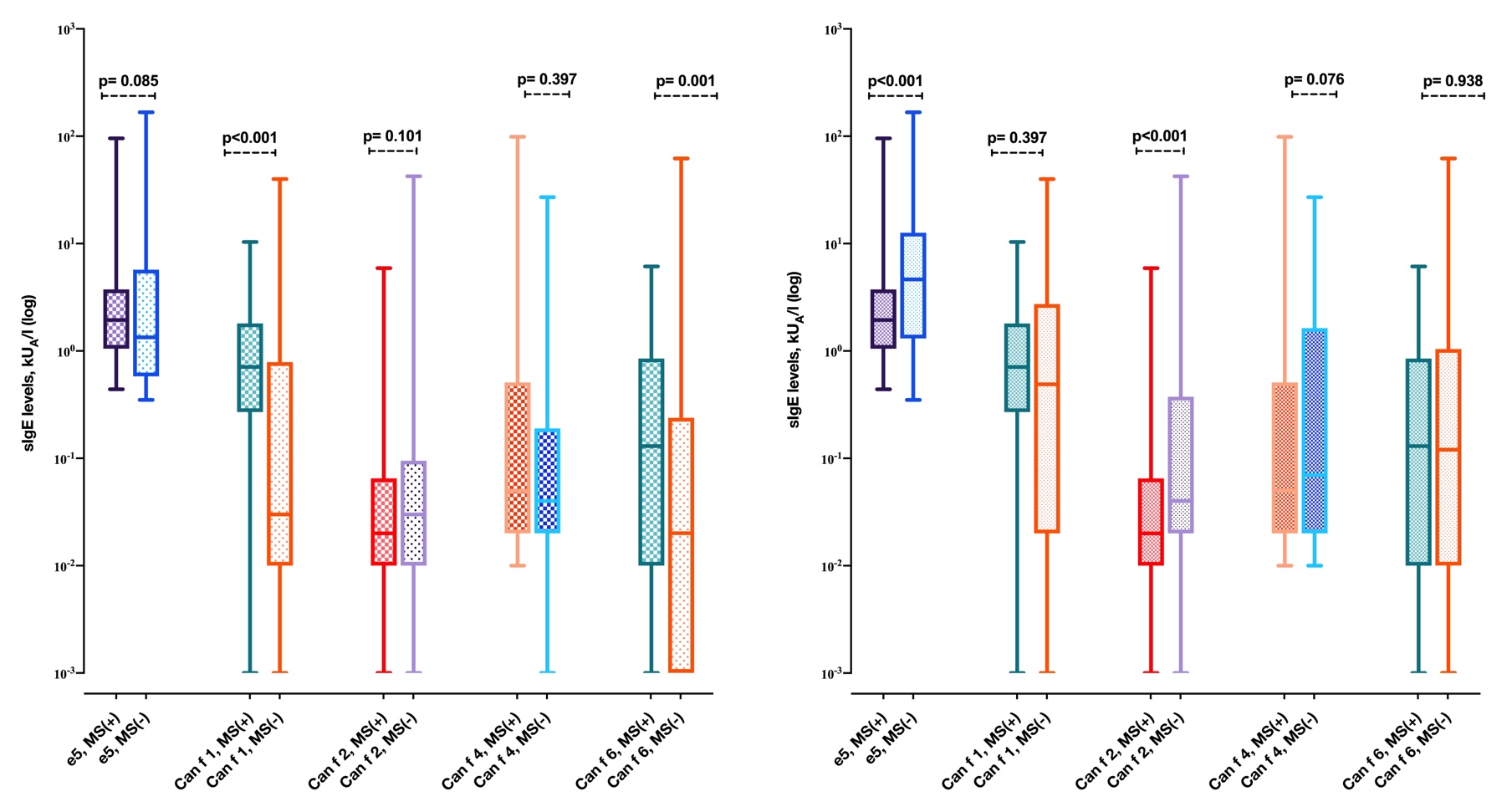
**

**Figure S6: Monosensitization to lipocalin show increased levels of sIgE to Can f 1 and Can f 6 in all study population while these results did not remain same among subjects sensitized to at least one dog allergen component. Comparison of** median sIgE level to each lipocalin by mono-sensitization (n=93) vs no mono-sensitization to lipocalins (n=220) in all study group **(Fig S6A**). Median of each lipocalin by mono-sensitization (n= 93) vs no mono-sensitization to lipocalins (n=125) among subjects sensitized to at least one dog allergen component **(Fig S6B).** e5= dog dander immunoglobulin E, sIgE= specific immunoglobulin E, MS= Monosensitization to lipocalins.

* Data are presented as median, and whiskers indicate the minimum and maximum values.

**A B**

**
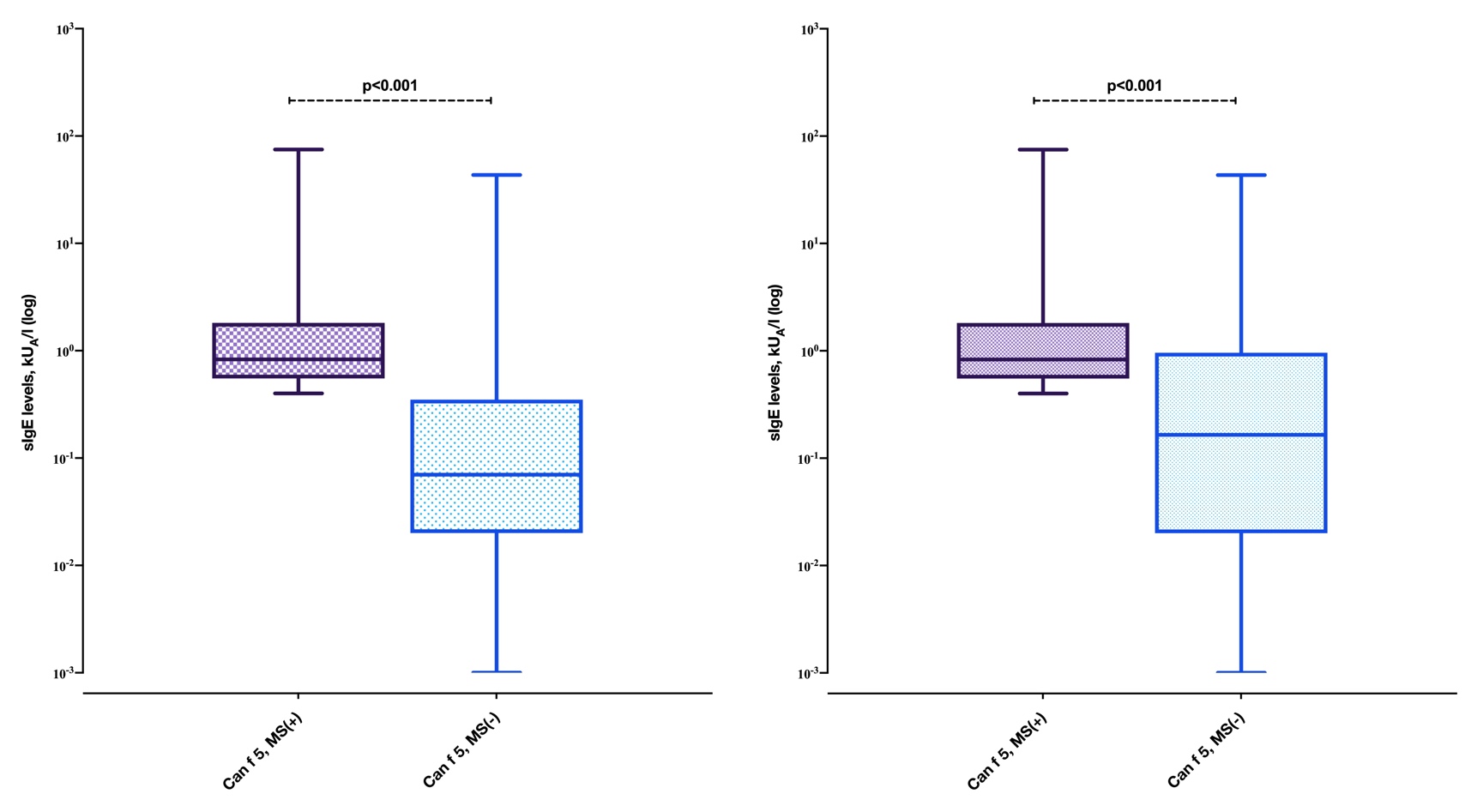
**

**Figure S7: Comparison of** median Can f 5 level by mono-sensitization(n=36) vs no mono-sensitization to prostatic kallikrein (n=277) in all study group **(Fig S7A).** Comparison of median Can f 5 level by mono-sensitization(n=36) vs no mono-sensitization to prostatic kallikrein (n=182) among subjects sensitized to at least one dog allergen component **(Fig S7B.** sIgE= serum immunoglobulin E, MS= Monosensitization to prostatic kallikrein.

* Data are presented as median, and whiskers indicate the minimum and maximum values.

**A B**

**
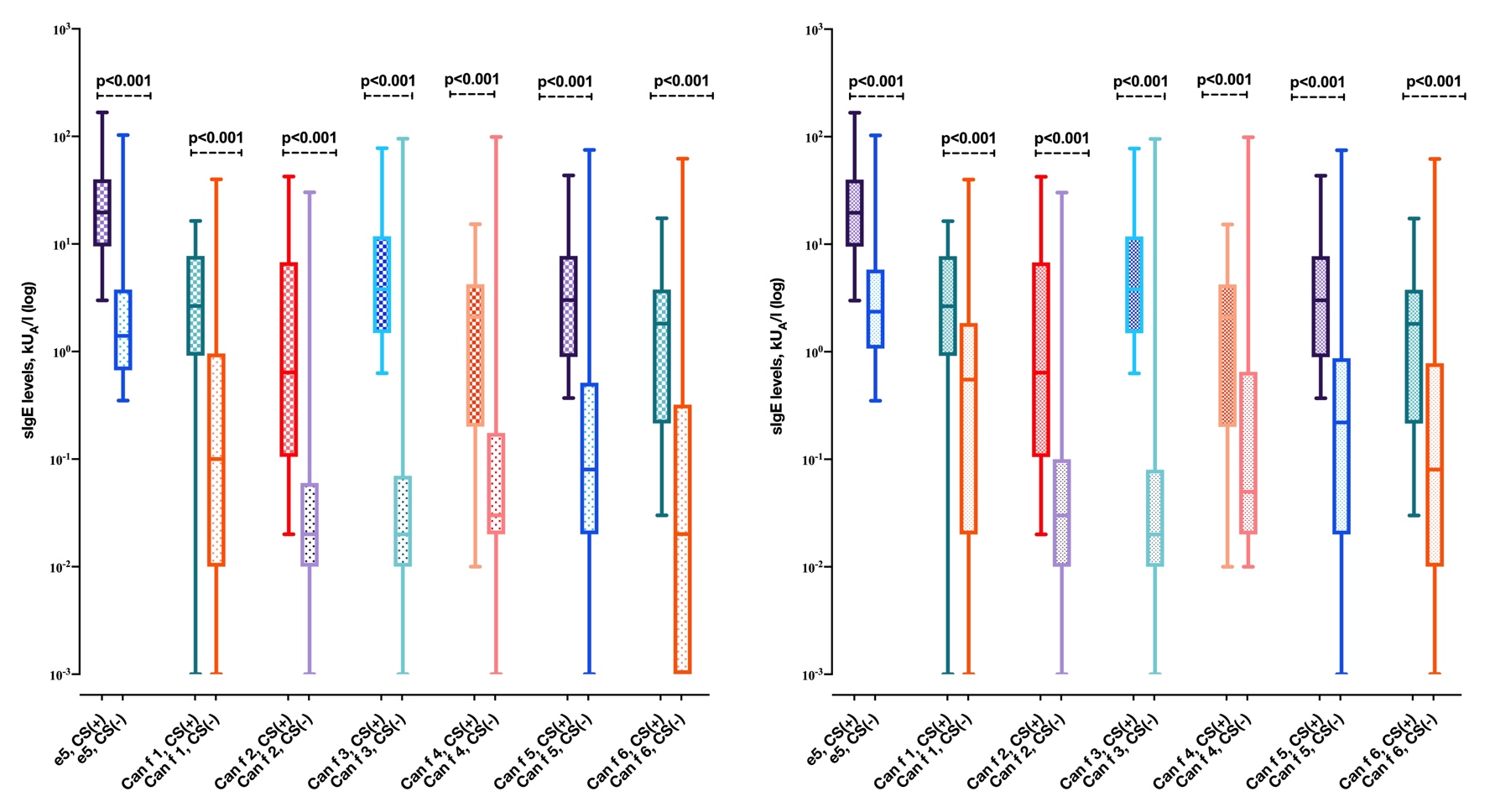
**

**Figure S8: Concomitant sensitization to lipocalins, albumin, and prostatic kallikrein show increased levels of sIgE for all subgroups.** Comparison of median sIgE levels to each dog allergen component by concomitant sensitization (n=21) vs non-concomitant sensitization (n=292) in all study group **(Fig S8A)**. Comparison of median sIgE levels to each dog allergen component by concomitant sensitization (n=21) vs non-concomitant sensitization (n=197) among subjects sensitized to at least one dog allergen component **(Fig 8B)**. CS= Concomitant sensitization, e5= dog dander immunoglobulin E, sIgE= serum immunoglobulin E.

* Data are presented as median, and whiskers indicate the minimum and maximum values.
