# Supplementary material for: Sensitization to molecular dog allergens in an adult population: Results from the West Sweden Asthma Study
Source: Clin Exp Allergy. 2022 Sep 1;53(1):88–104. doi: 10.1111/cea.14216 (PMC10087160; doi:10.1111/cea.14216)
Supplement: Supplementary file 2 — Table S1‐S2 [file CEA-53-88-s001.docx]

***SUPPORTING INFORMATION***

**Table S1:** Comparison of subjects sensitized to at least one dog allergen component and subjects not sensitized to any dog allergen component in relation to background characteristics

| Background characteristics | Frequency  *N* = 313  *n* (%) | Not sensitized to any dog component  *n=* 95 (30.4%)  *n (%)* | Sensitized to at least component ^†^  *n* =218 (69.6%)  *n* (%) | *P-value* |
| --- | --- | --- | --- | --- |
| Gender  Males  Females | 148 (47.3)  165 (52.7) | 50 (33.8)  45(27.3) | 98 (66.2)  120 (72.7) | 0.221 |
| Age, years  ≤30  31–45  46–60  61–75 | 74 (23.6)  103 (32.9)  96 (30.7)  40 (12.8) | 23 (31.1)  28 (27.2)  27 (28.1)  17 (42.5) | 51 (68.9)  75 (72.8)  69 (71.9)  23 (57.5) | 0.318 |
| Smoking status  Non-smokers  Ex-smokers  Current smokers  Missing information | 194 (62.0)  71 (22.7)  47 (15.0)  1 (0.3) | 65 (33.5)  17 (23.9)  13 (27.7) | 129 (66.5)  54 (76.1)  34 (72.3) | 0.294 |
| BMI, kg/m^2^  <25  25–29.9  ≥30 | 123 (39.3)  127 (40.6)  63 (20.1) | 40 (32.5)  36 (28.3)  19 (30.2) | 83 (67.5)  91 (71.7)  44 (69.8) | 0.772 |
| Exposure to dust/fumes at workplace  No  Yes  Missing information | 233 (74.4)  75 (24.0)  5 (1.6) | 64 (27.5)  29 (38.7)  2 (40.0) | 169 (72.5)  46 (61.3)  3 (60.0) | 0.066 |
| Raise on a farm  No  Yes  Missing information | 290 (92.7)  19 (6.1)  4 (1.3) | 87 (30.0)  7 (36.8)  1 (25.0) | 203 (70.0)  12 (63.2)  3 (75.0) | 0.530 |
| Degree urbanization  >10 000 inhabitants  ≤10 000 inhabitants | 225 (71.9)  88 (28.1) | 69 (30.7)  26 (29.5) | 156 (69.3)  62 (70.5) | 0.846 |
| Highest education attained  Less than high school  High school  Tertiary | 34 (10.9)  129 (41.2)  150 (47.9) | 13 (38.2)  32 (24.8)  50 (33.3) | 21 (61.8)  97 (75.2)  100 (66.7) | 0.173 |
| Family history of allergy or asthma  No  Yes | 111 (35.5)  202 (64.5) | 34 (30.6)  61 (30.2) | 77 (69.4)  141 (69.8) | 0.937 |
| Currently owns a dog  No  Yes | 267 (85.3)  46 (14.7) | 84 (31.5)  11 (23.9) | 183 (68.5)  35 (76.1) | 0.304 |
| Current asthma  No  Yes | 92 (29.4)  221 (70.6) | 34 (37.0)  61 (27.6) | 58 (63.0)  160 (72.4) | 0.101 |
| Current asthma and allergic rhinitis    No  Yes | 134 (42.8)  179 (57.2) | 50 (37.3)  45 (25.1) | 84 (62.7)  134 (74.9) | 0.020 |
| Current asthma without allergic rhinitis  No  Yes | 271 (86.6)  42 (13.4) | 79 (29.2)  16 (38.1) | 192 (70.8)  26 (61.9) | 0.241 |
| Current allergic rhinitis  No  Yes | 68 (21.7)  245 (78.3) | 24 (35.3)  71 (29.0) | 44 (64.7)  174 (71.0) | 0.316 |
| Current allergic rhinitis without asthma  No  Yes | 247 (78.9)  66 (21.1) | 69 (27.9)  26 (39.4) | 178 (72.1)  40 (60.6) | 0.072 |
| Current allergic rhinoconjunctivitis  No  Yes  Missing data | 29 (9.3)  216 (69.0)  68 (21.7) | 22 (10.1)  151 (69.3)  45 (20.6) | 7 (9.7)  65 (68.4)  23 (24.2) | 0.509 |

^†^Sensitized to any of Can f 1, Can f 2, Can f 3, Can f 4, Can f 5 and Can f 6.

**Table S2:** Comparison of subjects sensitized to at least one dog allergen component and subjects not sensitized to any dog allergen component in relation to age, body mass index, and sIgE values

| Background characteristic | All  *N* = 313  *n* (%) | Not sensitized to any dog component  *n=* 95 (30.4%)  *n (%)* | Sensitized to any component ^†^  *n* =218 (69.6%)  *n* (%) | *P-value* |
| --- | --- | --- | --- | --- |
| Age, mean ± SD | 42.97 ± 14.13 | 44.28 ± 15.39 | 42.40 ± 13.55 | 0.279 |
| BMI, kg/m^2^, mean ± SD | 26.33 ± 4.47 | 26.09 ± 4.47 | 26.43 ± 4.48 | 0.534 |
| sIgE values for e5, kU_A_/l, median (IQR) | 1.585 (0.700-4.806) | 0.615 (0.445-1.106) | 2.729 (1.136-7.819) | < 0.001 |
| sIgE values for Can f 1, kU_A_/l, median (IQR) | 0.147 (0.010-1.313) | 0.013 (0.004-0.043) | 0.684 (0.030-2.480) | < 0.001 |
| sIgE values for Can f 2, kU_A_/l, median (IQR) | 0.026 (0.012-0.077) | 0.018 (0.010- 0.033) | 0.031 (0.014-0.155) | < 0.001 |
| sIgE values for Can f 4, kU_A_/l, median (IQR) | 0.040 (0.020-0.280) | 0.020 (0.015-0.060) | 0.061 (0.020-0.795) | < 0.001 |
| sIgE values for Can f 6, kU_A_/l, median (IQR) | 0.030 (0.20-0.606) | 0.010 (0.000-0.094) | 0.121 (0.010-0.930) | < 0.001 |
| sIgE values for serum albumin, kU_A_/l, median (IQR) | 0.022 (0.010-0.102) | 0.017 (0.007-0.035) | 0.027 (0.011-0.256) | < 0.001 |
| sIgE values for prostatic kallikrein, kU_A_/l, median (IQR) | 0.119 (0.002-0.480) | 0.033 (0.013-0.094) | 0.295 (0.030-1.110) | <0.001 |

^†^Sensitized to any of Can f 1, Can f 2, Can f 3, Can f 4, Can f 5 and Can f 6. BMI= Body Mass Index, e5= dog dander immunoglobulin E, IQR= Interquartile range, SD= Standard Deviation. sIgE= Specific immunoglobulin E.

^*^Missing data was available for Can f 4 and Can f 6 (n=2).
